# Multicolor flow cytometry-based immunophenotyping for preclinical characterization of nanotechnology-based formulations: an insight into structure activity relationship and nanoparticle biocompatibility profiles

**DOI:** 10.3389/falgy.2023.1126012

**Published:** 2023-07-04

**Authors:** Hannah S. Newton, Jenny Zhang, Duncan Donohue, Ragi Unnithan, Edward Cedrone, Jie Xu, Alison Vermilya, Tyler Malys, Jeffrey D. Clogston, Marina A. Dobrovolskaia

**Affiliations:** ^1^ Nanotechnology Characterization Laboratory, Cancer Research Technology Program, Frederick National Laboratory for Cancer Research Sponsored by the National Cancer Institute, Frederick, MD, United States; ^2^ Agilent Technologies, Santa Clara, CA, United States; ^3^ Statistics Department, Frederick National Laboratory for Cancer Research Sponsored by the National Cancer Institute, Frederick, MD, United States

**Keywords:** nanomaterials, immunophenotyping, flow cytometry, lymphocytes, monocytes, nanoparticles

## Abstract

**Introduction:**

Immunophenotyping, which is the identification of immune cell subsets based on antigen expression, is an integral technique used to determine changes of cell composition and activation in various disease states or as a response to different stimuli. As nanoparticles are increasingly utilized for diagnostic and therapeutic applications, it is important to develop methodology that allows for the evaluation of their immunological impact. Therefore, the development of techniques such as immunophenotyping are desirable. Currently, the most common technique used to perform immunophenotyping is multicolor flow cytometry.

**Methods:**

We developed two distinct multicolor flow cytometry immunophenotyping panels which allow for the evaluation of the effects of nanoparticles on the composition and activation status of treated human peripheral blood mononuclear cells. These two panels assess the presence of various lymphoid and myeloid-derived cell populations as well as aspects of their activation statuses—including proliferation, adhesion, co-stimulation/presentation, and early activation—after treatment with controls or nanoparticles. To conduct assay performance qualification and determine the applicability of this method to preclinical characterization of nanoparticles, we used clinical-grade nanoformulations (AmBisome, Doxil and Feraheme) and research-grade PAMAM dendrimers of different sizes (G3, G4 and G5) and surface functionalities (amine-, carboxy- and hydroxy-).

**Results and Discussion:**

We found that formulations possessing intrinsic fluorescent properties (e.g., Doxil and AmBisome) interfere with accurate immunophenotyping; such interference may be partially overcome by dilution. In the absence of interference (e.g., in the case of dendrimers), nanoparticle size and surface functionalities determine their effects on the cells with large amine-terminated dendrimers being the most reactive.

## 1. Introduction

Nanomedicines are used in both therapeutics and diagnostics of various diseases, including cancer, infectious disease, autoinflammation and allergy, and can be used to deliver drugs to various cell types to promote immune stimulation, inhibition, or avoid the immune system altogether. Because of their vast applications, nanomaterials are composed of a wide array of materials and subsequently have various physicochemical properties such as size, shape, and zeta-potential (1, 2). While nanomedicines have overcome many limitations of conventional therapies, they still have their own unique limitations due to their own properties (3, 4). Nanomaterials have been extensively studied in complement activation related pseudoallergy and to a lesser degree in association with delayed-type hypersensitivity (5–10). These materials can cause unpredictable hypersensitivity reactions due to composition and, therefore, assays that can detect these reactions are needed (7, 8). The evidence is rising for nanomaterials' utility as immunomodulatory agents with potential to improve cancer immunotherapies and establish novel therapeutic approaches for immune-mediated disorders (11–16). Thus, there is an increasing need to characterize their efficacy and safety especially in terms of immune modulation and immunotoxicity (9, 17–19).

Thus, a technique such as immunophenotyping would aid in the characterization of immune cell modification caused by such nanomaterials (20). Immunophenotyping identifies immune cell subsets via their antigen expression and can be used to examine the treatment effects on cellular makeup and activity (21, 22). Many different analytical techniques can be used to perform immunophenotyping and the most common and broadly accessible technique is multicolor flow cytometry (22, 23).

Therefore, we established two distinct immunophenotyping panels to evaluate the effects of nanomaterials on a variety of cells in both the lymphoid and myeloid-derived populations including, but not limited to T cells, B cells, dendritic cells (DCs), natural killer (NK) cells, NK T cells, and monocytes as well as markers of activation, co-stimulation, and adhesion. Due to the limited number of fluorescent channels available for analysis at a time, the most abundant cell subsets and those with immediate interest to bionanotechnology research due to the prior knowledge of their involvement in the efficacy and safety of nanoformulations were prioritized over other cell subsets. Agonists known to activate these cell subsets were included as positive controls. Next, we used both clinical-grade and commercial research-grade nanoparticles to verify the performance and determine the applicability of this method to preclinical nanoparticle characterization.

## 2. Materials and methods

### 2.1. Reagents

The detailed procedure and commercial reagents are described in the Nanotechnology Characterization Laboratory (NCL) protocols ITA-37.1 and ITA-37.2 (24, 25). Phosphate Buffered Saline (PBS), RPMI-1640 media, fetal bovine serum (FBS), penicillin streptomycin solution, L-glutamine, Ficoll-Paque Premium were from GE Life Sciences (Marlborough, MA, USA). Hank's balanced salt solution (HBSS) was from Gibco (Gaithersburg, MD, USA). Acridine orange (AO)/propidium iodide (PI) staining solution was from Nexcelom Bioscience (Lawrence, MA, USA). Phytohemagglutinin (PHA-M) and Phorbol 12-myristate 13-acetate (PMA) were from Sigma-Aldrich (Burlington, MA, USA). Ionomycin was originally obtained from Sigma-Aldrich (Burlington, MA, USA) and later from STEMCELL Technologies (Vancouver, CA). Oligodeoxyribonucleotide, human TLR9 ligand (ODN2216) was from InvivoGen (San Diego, CA, USA). Polyamidoamine (PAMAM) dendrimers (Generations 3-5 (G3-G5) with amine (NH2), hydroxy (OH), or carboxylated (COOH, succinamic acid) surfaces) were from Dendritech (Midland, MI, USA). LAL Reagent Water (LWR) was from Associates of Cape Cod Incorporated (East Falmouth, MA, USA). Feraheme (AMAG Pharmaceuticals, Waltham, MA, USA), AmBisome (Astellas Pharma US, Northbrook, IL, USA) and Doxil (Baxter International, Inc., Deerfield, IL, USA) were obtained from the National Institutes of Health (NIH) pharmacy. NovoFlow, NovoRinse, NovoClean were from Agilent Technologies (Santa Clara, CA, USA). eBioscience Flow Cytometry Staining Buffer and UltraComp eBeads Plus Compensation Beads were from Invitrogen (Waltham, MA, USA). Anti-human mouse antibodies used for flow cytometry were obtained from BioLegend (San Diego, CA, USA) or Invitrogen (Waltham, MA, USA) (Tables 1, 2). Paraformaldehyde (PFA) 20% Solution was from Electron Microscopy Science (Hatfield, PA, USA).

**Table 1:** Labeling antibodies.

Fluorophore	Marker	Clone	Company	Concentration (µg/ml)	Catalog #	Panel Use
FITC	CD8a	HIT8a	BioLegend	200	300905 or 300906	1
FITC	CD56	5.1H11	BioLegend	200	362545 or 362546	2
PE	CD4	OKT4	BioLegend	100	317409 or 317410	1
PE	CD14	M5E2	BioLegend	200	301805 or 301806	2
PE-Cy7	CD19	HIB19	BioLegend	100	302215 or 302216	1, 2
APC	CCR4	L291H4	BioLegend	50	359407 or 359408	1
APC	CD123	6H6	BioLegend	100	306011 or 306012	2
AF700	CD45RA	HI100	Invitrogen	50	56-0458-42	1
AF700	CD54	HA58	BioLegend	400	353125 or 353126	2
APC-Fire 750	TCR-γδ	B1	BioLegend	400	331227 or 331228	1
APC-Fire 750	CD20	2H7	BioLegend	200	302357 or 302358	2
Pacific Blue (PacBlue)	CD45	2D1	BioLegend	100	368539 or 368540	1, 2
Zombie Aqua	Viability—Live/dead	Not Applicable	BioLegend	Not Available	423101 or 423102	1, 2
BV570	CD3	UCHT1	BioLegend	80	300435 or 300436	1, 2
BV650	CD25	BC96	BioLegend	100	302633 or 302634	1
BV650	CD69	FN50	BioLegend	50	310933 or 310934	2
BV711	CD154	24–31	BioLegend	100	310837 or 310838	1
BV785	CD127	A019D5	BioLegend	50	351329 or 351330	1
BV785	CD11c	3.9	BioLegend	160	301643 or 301644	2

Each row indicates a single labeling antibody (or dye) and specifies its conjugated marker and fluorophore, clone, company of distribution, concentration, catalog number as per indicated company, and which immunophenotyping panel contains each antibody.

**Table 2:** Isotype antibodies.

Fluorophore	Isotype	Clone	Company	Concentration (µg/ml)	Catalog #	Panel Use
FITC	Mouse IgG1, *κ*	MOPC-21	BioLegend	200	400109 or 400110	1, 2
PE	Mouse IgG2b, κ	MPC-11	BioLegend	200	400313 or 400314	1
PE	Mouse IgG2a, κ	MOPC-173	BioLegend	200	400211	2
PE-Cy7	Mouse IgG1, κ	MOPC-21	BioLegend	200	400125 or 400126	1, 2
APC	Mouse IgG1, κ	MOPC-21	BioLegend	200	400121 or 400122	1, 2
AF700	Mouse IgG2b κ	eBMG2b	Invitrogen	200	56-4732-80	1
AF700	Mouse IgG1, κ	MOPC-21	BioLegend	500	400143	2
APC-Fire 750	Mouse IgG1, κ	MOPC-21	BioLegend	200	400195 or 400196	1
APC-Fire 750	Mouse IgG2b, κ	MPC-11	BioLegend	200	400371 or 400372	2
PacBlue	Mouse IgG1, κ	MOPC-21	BioLegend	500	400131	1, 2
BV570	Mouse IgG1, κ	MOPC-21	BioLegend	100	400159 or 400160	1, 2
BV650	Mouse IgG1, κ	MOPC-21	BioLegend	100	400163 or 400164	1, 2
BV711	Mouse IgG1, κ	MOPC-21	BioLegend	100	400167 or 400168	1
BV785	Mouse IgG1, κ	MOPC-21	BioLegend	100	400169 or 400170	1, 2

Each row indicates a single isotype antibody and specifies its conjugated isotype, clone, and fluorophore, company of distribution, concentration, catalog number as per indicated company, and which immunophenotyping panel contains each antibody.

### 2.2. Nanoparticle preparation

#### 2.2.1. Dendrimers

Ethylenediamine (EDA) core, generation (G) three (3), four (4) and five (5), polyamidoamine (PAMAM) dendrimers with amine, hydroxy, or carboxylated surfaces (G5-COOH, G5-OH, G5-NH2, G4-NH2, or G3-NH2) were obtained from Dendritech Inc. as indicated in Section 2.1 and stored at 4°C. The hydroxy and amine dendrimers were provided in methanol while the carboxylated dendrimers were provided neat (lyophilized). Single vial aliquots of the hydroxy and amine dendrimers containing 1 mg of dendrimers were prepared as follows: Roughly 200 µl of sample was aliquoted into a pre-weighed Eppendorf tube. The methanolic solution was then entrained with nitrogen and lyophilized overnight. The lyophilized sample was then weighed, and the actual sample weight determined. To the known lyophilized sample, an appropriate volume of Milli-Q water was added to give a concentration of 10 mg/ml. 100 µl aliquots (corresponding to 1 mg of sample) were prepared from this stock sample solution, frozen, then lyophilized overnight to yield 1 mg aliquots. Single vial aliquots of the carboxylated dendrimers containing 1 mg of dendrimers were prepared by weighing an appropriate amount of dendrimer and adding the corresponding volume of Milli-Q water to give a concentration of 10 mg/ml. 100 µl aliquots (corresponding to 1 mg of sample) were prepared from this stock sample solution, frozen, then lyophilized overnight to yield 1 mg aliquots. All lyophilized samples were stored at −80°C before use. Prior to the experiment, lyophilized dendrimers were resuspended in 100 µl sterile water to obtain a final stock concentration of 10 mg/ml; these stock solutions could be kept at 4°C for up to 1 week. For the dendrimer samples, the stock solutions were diluted to 2 mg/ml using sterile water before addition to cells.

#### 2.2.2. Feraheme

Feraheme (30 mg/ml) was obtained from the NIH pharmacy as indicated in Section 2.1, stored at 4°C, and prepared prior to the start of the experiment. Stock Feraheme (30 mg/ml) was diluted to 10 mg/ml using 1× PBS as a dilutant each time Feraheme was needed. Feraheme (10 mg/ml) was added to cells at a minimal required dilution (MRD) of 10 for a final concentration of 1 mg/ml. Vehicle control for Feraheme was 1× PBS at MRD 10.

#### 2.2.3. AmBisome

Lyophilized AmBisome was obtained from the NIH pharmacy as indicated in Section 2.1 and prepared prior to the start of the experiment as according to the package insert (26). In brief, AmBisome (amphotericin B) liposome for injection was obtained as 50 mg lyophilized drug in a vial. An aliquot of 12 ml Sterile Water for Injection was added to the AmBisome vial (4 mg/ml amphotericin B). The vial was then shaken vigorously until the solution was translucent. Next, a working stock of AmBisome (1 mg/ml amphotericin B) was prepared by using 5% Dextrose Injection as the diluent and passing the solution through the filter provided with the package. This working stock (1 mg/ml amphotericin B) was stored at 4°C; dynamic light scattering and zeta potential were conducted once a week throughout the entire duration of the immunophenotyping analysis to confirm nanoparticles integrity (data not shown). On day of experiments, AmBisome was serially diluted from 1 mg/ml to 10 µg/ml. AmBisome (10 µg/ml amphotericin) was added to cell samples at the assay MRD 10 for a final concentration of 1 µg/ml. The vehicle control for AmBisome was 5% Dextrose Injection.

#### 2.2.4. Doxil

PEGylated liposomal doxorubicin (Doxil) was obtained from the NIH pharmacy as a solution with doxorubicin concentration of 2 mg/ml. The stock was stored at 4°C. On day of treatment, four dilutions of Doxil (0.2033, 0.0203, 0.0041, or 0.0008 mg/ml) were prepared using complete RPMI medium as the diluent. The various Doxil concentrations were added to the cells at assay MRD 5.

### 2.3. Physicochemical characterization

Size and surface chemistry of the dendrimers and AmBisome were assessed using dynamic light scattering (DLS) and zeta potential using NCL protocols PCC-1 and PCC-2 (27–29), respectively. Briefly, all dendrimer samples were prepared at 3 mg/ml in 10 mM NaCl for DLS and zeta potential measurements. In addition, the samples were also filtered through a 0.1 µm filter (Whatman Anotop 0.1) before measuring the hydrodynamic size. Zeta potential measurements were made at native pH as well as after pH adjustment to 7.4 using either standardized 0.1 M HCl or 0.1 M NaOH. Stock AmBisome was diluted 100-fold in 10 mM NaCl for DLS and zeta potential measurements. Zeta potential measurements were made at native pH as well as after pH adjustment to 7.4 using standardized 0.1 M NaOH. A Malvern Zetasizer Nano ZS instrument (Southborough, MA) with back scattering detector (173°) was used for measuring the hydrodynamic size (diameter) and zeta potential. Physicochemical characterization of Doxil and Feraheme was reported earlier (30–34).

### 2.4. Endotoxin detection

Endotoxin levels of dendrimer samples were evaluated using the Limulus Amebocyte Lysate (LAL) kinetic turbidity assay as per NCL protocol STE-1.2 (35). Briefly, endotoxin and lysate standards were prepared. The dendrimers were prepared in LWR at 10 mg/ml. A 4-point standard curve of endotoxin standard (1, 0.1, 0.01, 0.001 EU/ml) was prepared along with an endotoxin positive control (0.05 EU/ml). The negative control, standard curve, and positive control reactions were then run on the PyrosKinetix (Associates of Cape Cod Incorporated; East Falmouth, MA, USA). Diluted dendrimer samples (Dil 5, Dil 50, and Dil 500) and diluted dendrimer samples + endotoxin spike (0.05 EU/ml) were prepared, and reactions were run. Endotoxin in AmBisome, Doxil and Feraheme was not tested as these clinical-grade formulations contained certificate of analysis by the manufacturer.

### 2.5. Peripheral blood mononuclear cells (PBMC) isolation and culture

#### 2.5.1. Donor blood and PBMC isolation

Whole blood from healthy human donors was collected in BD Biosciences li-heparin vacutainers (San Jose, CA, USA) under National Cancer Institute (NCI)-Frederick protocol OH9-C-N046 and used for PBMC isolation as indicated in NCL protocol ITA-10 (36). In brief, the whole blood was diluted with PBS at equal volume. The diluted whole blood was layered over Ficoll-Paque Premium solution (3 ml Ficoll-Paque per 4 ml of diluted whole blood). The samples were then centrifuged for 30 min at room temperature at 900×*g* with no brake applied. The mononuclear cell layer was then collected in a new tube using a sterile pipette and washed twice with HBSS (centrifuged for 10 min at 400×*g* at room temperature). The cells were then resuspended in complete RPMI-1640 medium (10% heat inactivated FBS, 100 U/ml penicillin, 100 µg/ml streptomycin, and 2 mM L-glutamine) and counted on a Cellometer using a 1:1 ratio of cell suspension to AOPI. Once the cells were counted, samples were diluted to 1.25 × 10^6^ cells/ml.

#### 2.5.2. PBMC activation and culture with nanoparticles

PBMC were cultured in 24-well plates at a final concentration of 1 × 10^6^ cells/ml (1 ml total) in a 37°C/95% CO_2_ incubator for approximately 24 h. Unstimulated PBMC were used as negative controls. PBMC were activated with 20 ng/ml LPS + 10 µg/ml PHA-M, 50 ng/ml PMA + 1 µg/ml Ionomycin, or 5 µg/ml ODN2216 + 10 µg/ml PHA-M. Additionally, PBMC were treated with 10 µg/ml dendrimers (G5-COOH, G5-OH, G5-NH2, G4-NH2, or G3-NH2), 1 µg/ml AmBisome (amphotericin B), 1 mg/ml Feraheme, or respective vehicle controls and cultured for 24 h. Complete RPMI-1640 medium was added to cell samples to obtain a final volume of 1 ml. All concentrations indicated are final concentrations.

### 2.6. Multicolor flow cytometry

#### 2.6.1. Antibody titration

Antibody titration was performed as described in NCL protocol ITA-37.1 using both compensation beads and PBMC (24). In brief, the antibody/dye titration was performed as a six-point calibration curve [stock, dilution (Dil) 5, Dil 25, Dil 125, Dil 625, Dil 3,125] for each antibody used in the immunophenotyping panels. First, a 10-fold dilution of UltraComp eBeads Plus compensation beads was made with staining buffer and 50 µl of diluted beads were added to 96-well plate wells (one well per antibody dilution). An aliquot of 50 µl PBMC (50% live/50% dead) at approximately 5 × 10^5^ cells/ml was added to six empty wells—needed for the Zombie Aqua viability dye. The six-point antibody/dye titrations (serial dilutions) were prepared—using staining buffer as the diluent for the antibodies and 1× PBS as the diluent for the viability dye. An aliquot of 5 µl antibody/dye dilution was added to the wells containing the 50 µl beads or PBMC (MRD of approximately 10)—one antibody/dye dilution per 50 µl beads/PBMC. The plate was then incubated at room temperature for 30 min in the dark. The plate was then centrifuged for 1–3 min at 300×*g* and the supernatant was removed (leaving a small residual volume). The samples were then resuspended in staining buffer for a total of about 50 µl.

The samples were then run on the NovoCyte 3005 (Agilent; Santa Clara, CA). The necessary parameters were selected as indicated in NCL protocol ITA-37.1 (24). At least 12,000 events/30 µl were collected per sample and the file was saved. Data analysis was then performed in NovoExpress. For each sample, a plot with forward (FSC) and side (SSC) scatter was made to allow for a region of interest to be drawn around the beads (or PBMC). A histogram was then made with the appropriate fluorophore on the x-axis and count on the y-axis and all the serial dilutions of a single antibody were plotted on the same histogram.

#### 2.6.2. Single stain controls

Single stain controls were prepared as indicated in NCL protocol ITA-37.1 (24). In brief, the optimal antibody titrations as determined by the antibody titration described in section 2.6.1 were used to perform single stain controls. Single stain controls were run for each panel every time a new antibody lot was obtained. A 10-fold dilution of UltraComp eBeads Plus compensation beads was made with staining buffer and 50 µl of diluted beads were added to 96-well plate wells (one well per antibody). An aliquot of 50 µl PBMC (50% live/50% dead) at approximately 5 × 10^5^ cells/ml was added to 2 wells (for viability dye). Prepared at least 5 µl of optimal antibody/dye titration as previously determined and added 5 µl of antibody/dye dilution to corresponding well with beads or PBMC. Incubated the samples in plate in the dark for 30 min at room temperature. Centrifuged the plate for 5 min at 300×*g* and aspirated the supernatant. Resuspended the samples in 50 µl staining buffer.

The samples were then run on the NovoCyte 3005. The necessary parameters were selected as indicated in NCL protocol ITA-37.1 using the auto compensation setting in the NovoCyte software (24). At least 25,000 events/50 µl were collected per sample and the file was saved.

#### 2.6.3. Fluorescence minus one (FMO) controls

FMOs were prepared as indicated in NCL protocol ITA-37.1 (24). In brief, activated PBMC (prepared as indicated in section 2.5) were used for the samples. The samples needed for FMOs are unstained, fully stained (all labeling antibodies), and an FMO for each antibody/dye (sample containing all antibodies/dyes except one) and were run every time a new antibody lot was obtained. PBMC to be stained with the antibodies/dye needed to have live: dead ratio of 3:1. A sufficient volume of activated PBMC were centrifuged for 10 min at 400×*g* resuspended in 1× PBS, and heat shocked at 70°C for at least 1.5 h. The remaining live PBMC samples were centrifuged for 10 min at 400×*g* resuspended in 1× PBS and combined with the heat shocked (dead) PBMC at a ratio of 3:1. Centrifuged the samples for 7 min at 400×*g* and resuspended each sample in 49.2 µl 1× PBS. Added 0.8 µl of 5-fold diluted Zombie Aqua dye to all samples except the unstained and Zombie Aqua FMO sample. Incubated samples for 30 min at room temperature in the dark. Next, each sample was washed twice (400×*g* for 7 min) with staining buffer. The samples were then resuspended in 40 µl staining buffer. Master mixes for each necessary FMO and stained samples were prepared as indicated in NCL protocol ITA-37.1 (24). The antibodies were added to each master mix at the volume that corresponded to the optimal titration dilution described in 2.6.1. The appropriate master mix was added to the corresponding cell sample and the samples were incubated for 30 min at room temperature in the dark. The samples were then washed with staining buffer and fixed with 100 µl 2% PFA for 15 min. The samples were then washed twice with staining buffer and resuspended in 500 µl staining buffer.

The Novocyte 3005 was used to perform data acquisition. The parameters (channels and laser intensities) used for this acquisition were the same as the parameters used in the single stain controls and are indicated in NCL protocol ITA-37.1. At least 300 µl were collected per sample. Data analysis was performed in NovoExpress and described in NCL protocol ITA-37.1 (24). The compensation matrix from the single control samples was applied to the FMO control experimental file. A plot was made for each FMO sample and gates were made to eliminate debris and doublet cells. The compensation was then adjusted using the fully stained sample with FMO sample overlays to account for the differences between compensation beads and cell samples. The adjusted compensation matrices were then saved and used for the immunophenotyping panels.

#### 2.6.4. Flow cytometry with Doxil-treated PBMC

Some control experiments were run on the NovoCyte 3005 without staining antibodies. In these experiments, PBMC (0.2 × 10^6^ cells) were treated with varying concentrations of Doxil (0.2033, 0.0203, 0.0041, or 0.0008 mg/ml) (MRD 5; 200 µl total volume) overnight in the 37°C/95% CO_2_ incubator. The PBMC were then washed with staining buffer consisting of 1× PBS + 2% FBS and then fixed with 1%–2% PFA. The fixed samples, devoid of any staining antibodies, were then acquired on the NovoCyte 3005 with the same parameters previously determined.

#### 2.6.5. Immunophenotyping panels

The procedure used for running the immunophenotyping panels is described in length in NCL protocol ITA-37.2 (25). Unstimulated (negative control), activated (positive controls), and treated PBMC as prepared in section 2.5 were used to run two panels—Immunophenotyping Panel #1 and Immunophenotyping Panel #2. PBMC were activated with LPS/PHA-M, PMA/Ionomycin, or ODN2216/PHA-M as indicated in section 2.5 or were treated with dendrimers, AmBisome, Feraheme or vehicle controls for approximately 24 h as indicated in section 2.5. Next, the master mixes for each antibody panel were prepared using the previously determined optimal antibody titrations for each individual antibody. Each panel had a labeling antibody master mix and an isotype control master mix with each isotype antibody concentration matching the final concentration of the corresponding labeling antibody. The cell samples were then centrifuged at 400×*g* for 10 min. The cells were then washed 2× with 1× PBS and resuspended in 49.2 µl 1× PBS. The appropriate samples (all fully stained labeling antibodies of both panels) were stained with 0.8 µl of Zombie Aqua dye Dil 5 for 30 min at room temperature in the dark. The samples were then washed twice with staining buffer that contained FBS to decrease non-specific binding and resuspended in appropriate amount of staining buffer (for final volume of 100 µl). Next, the appropriate amount of antibody master mix was added to each appropriate sample and incubated for 30 min at room temperature in the dark. Finally, the samples were washed with staining buffer; fixed for 15 min at room temperature using 100 µl 2% PFA; washed twice more with staining buffer; resuspended with 500 µl staining buffer; and stored at 4°C before subsequent data acquisition.

Data acquisition was performed on the NovoCyte 3005. The parameters were the same as the single stain control and FMO control parameters. The collection settings were set to at least 500,000 events—some conditions had fewer total events collected. The data was then saved, and the compensation matrix obtained from the FMO controls was applied to each data file. The FCS files were then uploaded to FCS Express 7 and analyzed. A detailed step by step description of the gating process is in NCL protocol ITA-37.2 (25). Isotype control samples were used as the negative staining controls to set the gate parameters for each activation or treatment condition—i.e., unstimulated isotype controls were used to set the gates for all unstimulated stained samples while activated isotype controls were used to set the gates for all activated stained samples. These isotype controls aid in controlling for non-specific binding—including non-specific Fc receptor binding. For data analysis, the stimulation index (or fold change) of (1) gate percentages and (2) geometric mean fluorescence intensities (gMFIs) of the different samples were determined by comparison to the unstimulated (negative) control values. Microsoft Excel, R programming language, and Graph Pad Prism were used for data analysis and visualization.

### 2.7. Statistical analysis

Statistical analysis was performed on the gate percentage values. Technical repeat and negative control variability was estimated using Pearson's correlation of gate percentages across all negative control technical repeat pairs. Complete agglomeration clustering on the Euclidean distance matrix of Pearson's R values, as implemented in the heatmap.2 package of the gplots package for R (https://CRAN.R-project.org/package=gplots), was used to cluster technical repeat control runs. Comparison of treatment groups was performed on technical repeat averaged gate percentage values. Due to differences between untreated controls and vehicle controls, vehicle controls were used as the reference group where available (Dextrose and PBS for AmBisome and Feraheme, respectively). For each gate, nanoparticle treatment groups were compared to their respective controls using a paired *t*-test. To account for any test bias and to control for family-wise error rates, the resulting *t*-test *p*-values were corrected using a null hypothesis distribution of permuted *p*-values. Implemented in a custom R script, treated/control labels were swapped for donor pairings to create exhaustive unique permutations of the data while keeping donor pairing and gate correlation structure intact. All individual paired *t*-test *p*-values were compared to this null hypothesis distribution to generate permutation *p*-values using previously described algorithm (37).

## 3. Results

### 3.1. Development of immunophenotyping panels

Immunophenotyping uses antigen expression to define cellular subsets. However, the antigens and gating strategies needed to define subsets vary according to different research groups. Our goal was to develop a series of multicolor flow cytometry panels which could be used to define a broad spectrum of lymphoid and myeloid-derived immune cell subsets (and their activation statuses) pre- and post-nanomaterial treatment which could be adopted by various research laboratories.

The initial steps of the multicolor flow cytometry immunophenotyping panel development are (1) defining the desired cell types and activation markers and (2) determining the suitable fluorophore conjugates based upon the cytometer capabilities. Once we defined desired cell types, we used FluoroFinder and other spectral builders such as ThermoFisher SpectraViewer to determine suitable conjugates and potential spectral overlap (38, 39). In silico panel development led to two distinct panels.

We named the first panel *Immunophenotyping Panel #1 (or Lymphocyte Panel).* This panel included antibody-fluorophore conjugates that allow for analysis of different lymphocyte populations including B cells and T cells [CD8^+^ T cells, CD4^+^ T cells, regulatory *T* (*T*_reg_) cells, naïve T cells, and γδ TCR T cells] (Table 3). This panel also determined cellular CD25 and CD154 expression which are markers of activation suggesting proliferation and co-stimulation/presentation, respectively (Table 3).

**Table 3:** Immunophenotyping panel #1 (or *lymphocyte panel*) markers.

Cell Population	Antibody/Dye—markers used for cell population definition					
B cells*	Viability (live)—Zombie Aqua-	CD45—Pacific Blue+	CD19—PE-Cy7+			
T cells	Viability (live)—Zombie Aqua-	CD45—Pacific Blue+	CD3—BV570+			
Cytotoxic T cells*	Viability (live)—Zombie Aqua-	CD45—Pacific Blue+	CD3—BV570+	CD8–FITC+/CD4–PE-		
Naïve Cytotoxic T cells	Viability (live)—Zombie Aqua-	CD45—Pacific Blue+	CD3—BV570+	CD8–FITC+/CD4–PE-	CD45RA—AF700+	
CD4 T cells*	Viability (live)—Zombie Aqua-	CD45—Pacific Blue+	CD3—BV570+	CD4—PE+/CD8a—FITC-		
Regulatory T cells	Viability (live)—Zombie Aqua-	CD45—Pacific Blue+	CD3—BV570+	CD4—PE+/CD8a—FITC-	CCR4—APC+	CD25—BV650+/ CD127—BV785 lo
TCR g/d T cells	Viability (live)—Zombie Aqua-	CD45—Pacific Blue+	CD3—BV570+	CD8a—FITC-/CD4—PE-	delta +	
*Activation markers	CD25—BV650	BV711—CD154				

Each cell population is defined by a certain set of antibodies. Cell populations with an asterisk (*) were also evaluated for activation markers CD25 and CD154.

We named the second panel *Immunophenotyping Panel #2 (or Monocyte, Dendritic cell (DC), Natural Killer (NK) cell Panel).* Panel 2 included antibody-fluorophore conjugates that allow for analysis of different cell populations including CD14^+^ monocytes, DCs (plasmacytoid (p) and myeloid (m) DCs), and NK cells along with NK T cells (Table 4). In terms of activation, this panel assessed cellular CD69 and CD54 expression which are markers of early activation and adhesion, respectively (Table 4). Once panel development was complete, the panels were qualified using positive controls as detailed further below in section 3.3.

**Table 4:** Immunophenotyping panel #2 (or monocyte, dendritic cell (DC), natural killer (NK) cell panel) markers.

Cell Population	Antibody/Dye—markers used for cell population definition						
Monocytes*	Viability (live)—Zombie Aqua-	CD45—Pacific Blue+	CD3—BV570-	CD19—PE-Cy7-	CD14—PE +		
pDCs*	Viability (live)—Zombie Aqua-	CD45—Pacific Blue+	CD3—BV570-	CD19—PE-Cy7-	CD14—PE-	APC-Fire750—CD20-	CD123—APC+
mDCs*	Viability (live)—Zombie Aqua-	CD45—Pacific Blue+	CD3—BV570-	CD19—PE-Cy7-	CD14—PE-	APC-Fire750—CD20-	CD11c—BV785+
NK cells*	Viability (live)—Zombie Aqua-	CD45—Pacific Blue+	CD3—BV570-	CD19—PE-Cy7-	CD14—PE-	APC-Fire750—CD20-	CD56—FITC+
NK T cells	Viability (live)—Zombie Aqua-	CD45—Pacific Blue+	CD3—BV570+	CD56—FITC+			
*Activation markers	CD54—AF700	CD69—BV650					

Each cell population is defined by a certain set of antibodies. Cell populations with an asterisk (*) were also evaluated for activation markers CD54 and CD69.

### 3.2. Antibody titration and compensation of immunophenotyping panels

To run the immunophenotyping panels, there are many controls that need to be conducted including antibody/dye titrations, single stain compensation, and FMO controls. While commercially available antibodies usually have recommended test concentrations, it is important to titrate each antibody to determine the best concentration for the settings used in each individual experiment. To do this, we prepared a six-point serial dilution (stock, Dil 5, Dil 25, Dil 125, Dil 625, Dil 3,125) of each antibody/dye and used the titrations to stain compensation beads (or PBMC) with an additional MRD of approximately 10. Antibody titrations are represented in both visual (histograms) and numerical (stain index calculated in NovoExpress) formats (Supplementary Figure S1). Based on this data, the following optimal antibody dilutions from the titrations were determined for Immunophenotyping Panel #1—CD8-FITC Dil 25, CD4-PE Dil 125, CD19-PE-Cy7 Dil 125, CCR4-APC Dil 125, CD45RA-AF700 Dil 25, TCR-γδ-APC-Fire750 Dil 50, CD45-PacBlue Dil 25, CD3-BV570 Dil 25, CD25-BV650 Dil 100, CD154-BV711 Dil 50, CD127-BV785 Dil 25, and Viability (live/dead)-Zombie Aqua Dil 125 (Supplementary Figures S1A,C). Supplementary Figure S1B shows the antibody titrations for Immunophenotyping Panel #2. If a particular antibody was used in both immunophenotyping panels, the titration for that antibody (e.g. CD45-PacBlue) was not repeated during Panel #2 titrations. Instead, the optimal dilution for that antibody determined by Panel #1 titrations was used for both panels. Based on the titrations for Immunophenotyping Panel #2, the following dilutions were determined—CD56-FITC Dil 125, CD14-PE Dil 125, CD123-APC Dil 25, CD54-AF700 Dil 125, CD20-APC-Fire750 Dil 25, CD69-BV650 Dil 25, CD11c-BV785 Dil 25 (Supplementary Figures S1B,C).

Next, we ran single stain compensations to obtain compensation and spillover matrices. Using the optimal antibody dilutions, we stained compensation beads (or PBMC) with a single antibody (or dye). Single stain samples for each antibody/dye in each panel were run on the NovoCyte 3005 using the auto compensation function which automatically corrects spectral overlap for each fluorophore. Examples of single stain compensation plots for Immunophenotyping Panel #1 and Panel #2 are indicated in Supplementary Figures S2, S3.

Next, the compensation determined via single stain controls was verified and updated using activated PBMC for FMO controls. PMA/Ionomycin was used to activate cells for Immunophenotyping Panel #1 and ODN2216/PHA-M used to activate cells for Immunophenotyping Panel #2. Each FMO control was run, and the compensation matrix initially obtained from the single stain controls was adjusted to correct any over- or under-compensation. An example FMO control for each panel is indicated in Supplementary Figures S4, S5. The compensation matrices for Immunophenotyping Panel #1 and Panel #2 were then used to compensate the data acquired going forward. Each time a new antibody lot was obtained, the single stain and FMO compensations were redone to account for any inter-lot variations.

### 3.3. Positive controls validated immunophenotyping panels

**Figure 1:**
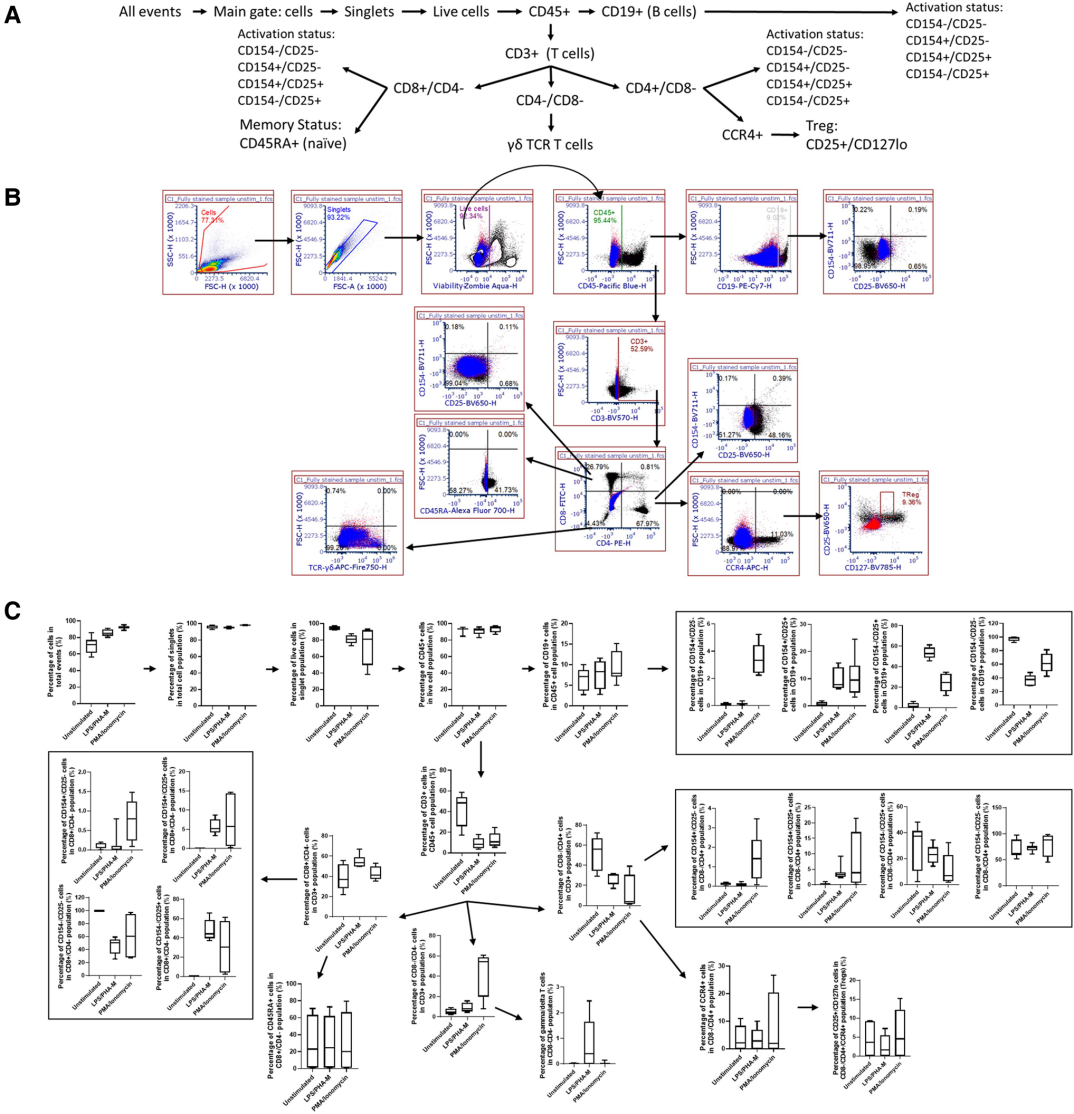
Gating strategy and validation of immunophenotyping panel #1 (lymphocyte panel). (**A**) This gating strategy was used to define certain cell populations. A gate for main cells was used on all events to exclude debris and then cells were gated to obtain singlet cells. A viability dye was then used to exclude dead cells and thus the live cells were selected. The live cells were then gated with CD45. CD19^+^ gate was then used to define the B cell population within the CD45^+^ gate and the markers CD154 and CD25 were used to determine the activation statuses of the CD19^+^ population. The CD45^+^ gate was also used to then select the CD3^+^ cell population (T cells). This CD3^+^ population was then gated on CD8 and CD4 markers. CD8^+^/CD4^−^ population defined cytotoxic T cells which were then further gated on naïve status (CD45RA+) and activation status (CD154/CD25). CD4^+^/CD8^−^ population defined helper T cells and their activation status (CD154/CD25) and was also used to further gate on CCR4^+^, CD25^+^, CD127lo cells to define *T*_regs_. The CD4^−^/CD8^−^ gate was gated on γδ TCR to define this population of T cells. (**B**) Gating shown for unstimulated PBMC from one replicate of healthy donor M4W2. Red and blue overlays are the isotype controls while the black overlay is a replicate of the fully stained PBMC with labeling antibodies. Each gate is an inclusive gate (cells in gate are included in further gating) except the viability (live/dead) gate which is an exclusive gate (cells in gate are excluded from further gating) (**C**) PBMC from four healthy donors (*N* = 4, 2 technical replicates per donor) were activated with positive controls (LPS/PHA-M or PMA/Ionomycin) or left unstimulated/untreated for 24 h. The percentage of cells in each gate for each donor were determined following the same gating strategy as shown in A. The percentages indicate the percentage of selected cells in parent gate rather than in the total events. Each box plot represents data of 2 replicates from each of four donors (M4W2, F4Z5, J7X3, E3T5). The middle bar is the median and the whiskers are the 10th and 90th percentiles of the data.

To determine whether the panels could detect the desired cell populations *in vitro*, the antibody panels defined in Table 1 were used to stain unstimulated and activated PBMC from three to four healthy donors at the optimal concentrations previously determined by antibody titrations. As controls for the labeled antibodies, samples of unstimulated and activated PBMC were left unstained or were stained with the appropriate isotype control antibodies as indicated in Table 2. For Immunophenotyping Panel #1, PBMC were activated with two different sets of positive controls—LPS/PHA-M and PMA/Ionomycin. Two different positive controls were used because the PMA/Ionomycin was better at increasing expression of CD154 (data not shown) than LPS/PHA-M, while LPS/PHA-M is a less harsh method of activation. Once the panel data was acquired on the NovoCyte 3005, the data was compensated using the compensation matrix determined from the FMO controls for Immunophenotyping Panel #1 and then analyzed in FCS express. The different cell populations defined in Table 3 were gated following the process outlined in Figure 1A. When defining the gates in FCS Express, the negative gates were set by the isotype control for each condition (Figure 1B). It was determined that the cell populations defined in Table 3 were successfully detected in the donor PBMC based on the gating strategy used (Figure 1C). Additionally, successful cell activation (or increases in CD25 and/or CD154) were observed in the indicated populations upon treatment with positive controls (Figure 1C). There was inter-donor variation for certain cell populations such as naïve CD8^+^ T cells, *T*_regs_, as well as for the activated cell populations (Figure 1C). Additionally, cell subsets such as naïve CD8^+^ T cells, *T*_regs_, and γδ TCR T cells have extremely small percentages of positive cells, thus careful consideration needs to be given when assessing stimulation index of these populations (Figure 1C).

**Figure 2:**
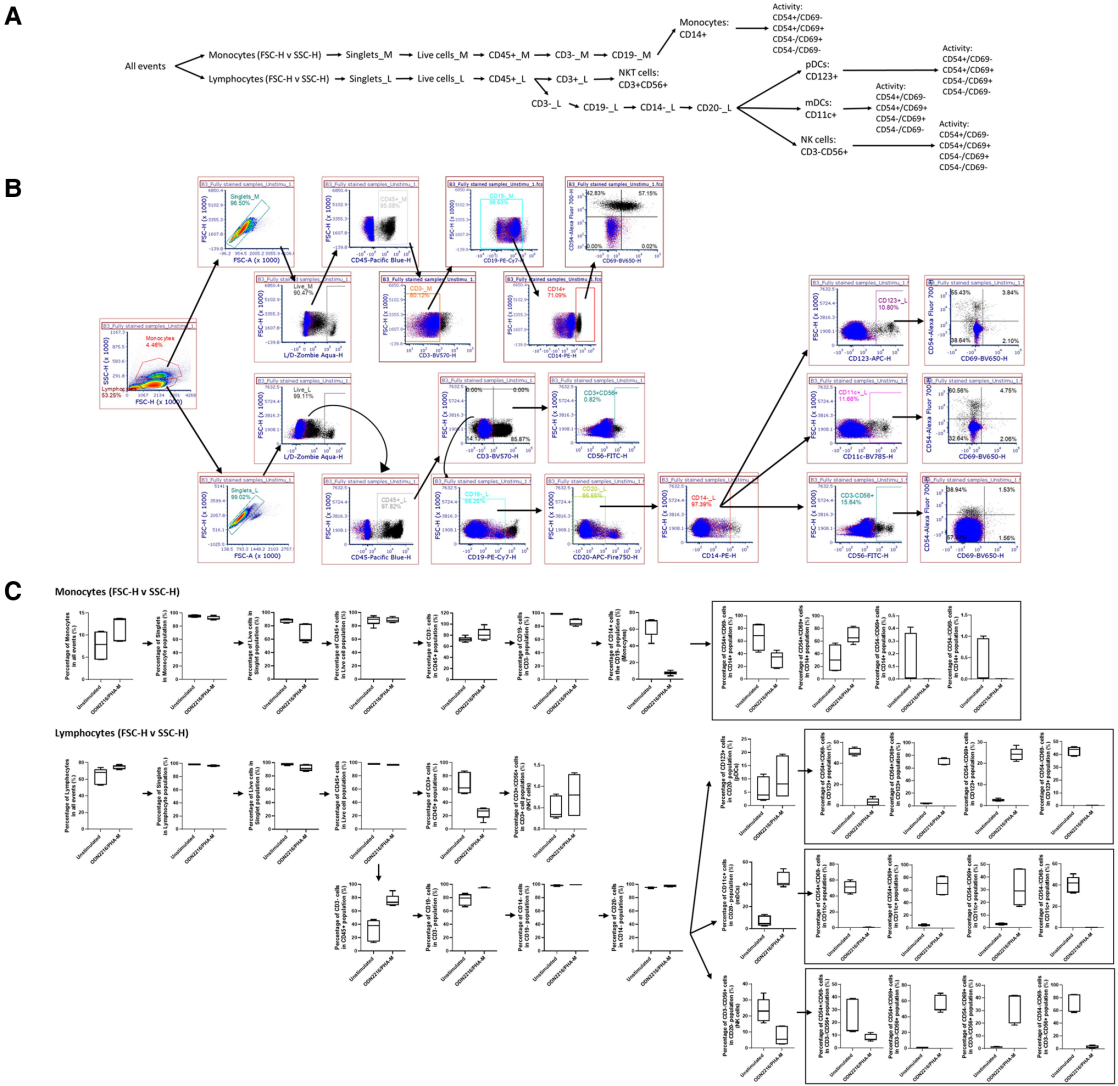
Gating strategy and validation of immunophenotyping panel #2 (monocyte, dendritic cell (DC), natural killer (NK) cell panel). (**A**) The gating strategy was used to define selected cell populations. FSC versus SSC gates were initially used to separate monocytes and lymphocytes based upon the morphology. Cells were gated to obtain singlet cells. A viability dye was then used to exclude dead cells. The live cells were then gated with CD45. In the monocyte population, the CD45^+^ cells were then gated on CD3^−^, CD19^−^ and CD14^+^ to obtain the CD14^+^ monocyte population. Activation status of these cells was determined by CD54/CD69 gates. In the lymphocyte population, the CD45^+^ cells were of each gated on CD3. CD3^+^ CD56^+^ cells indicated NK T cells. CD3^−^ cell population was gated on CD19^−^, CD14^−^, CD20^−^. From this gate, the CD123^+^ gate was used to define pDC. The CD11c^+^ gate was used to define mDCs, and CD56^+^ was used to define NK cells. Activation status of each of these cell populations was defined with CD54/CD69. (**B**) Gating shown for unstimulated PBMC from one replicate of healthy donor I5F3. Red and blue overlays are the isotype controls while the black overlay is a replicate of the fully stained PBMC with labeling antibodies. Each gate is an inclusive gate (cells in gate are included in further gating) except the viability (live/dead) gate which is an exclusive gate (cells in gate are excluded from further gating). (**C**) PBMC from three healthy donors (*N* = 3, 2 technical replicates) were activated with positive controls (ODN2216/PHA-M) or left unstimulated/untreated for 24 h. The percentage of cells in each gate for each donor were determined following the same gating strategy as shown in A. The percentages indicate the percentage of selected cells in parent gate rather than in the total events. Each box plot represents data of 2 replicates from each of three donors (E3H8, O4Q7, I5F3). The middle bar is the median and the whiskers are the 10th and 90th percentiles of the data.

For Immunophenotyping Panel #2, PBMC from three healthy donors were left unstimulated or were activated with ODN2216/PHA-M and stained with the appropriate antibodies (Tables 1, 2). The acquired data was then compensated using the previously acquired matrix from the FMO controls and gated based on the strategy depicted in Figure 2A to define the cell types defined in Table 4. The negative gates were set using the appropriate isotype controls (Figure 2B). The monocyte and lymphocyte populations defined with these antibodies were successfully identified (Figure 2C). The NKT cell population should be considered with caution as the percentage are minuscule (Figure 2C). ODN2216/PHA-M (positive control) also increased the percentage of cells positive for the activation markers CD54 and CD69 (Figure 2C). Once the assays were validated using known positive controls, the assays could be tested with different nanomaterials.

### 3.4. Doxil interfered with accurate assay performance

**Figure 3:**
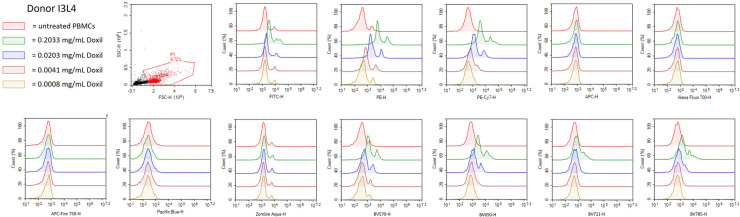
Doxil interferes with NovoCyte 3005 settings. PBMC from healthy donor I3L4 were treated with Doxil (0.2033 mg/ml, 0.0203 mg/ml, 0.0041 mg/ml, or 0.0008 mg/ml) or left untreated overnight and samples were fixed and acquired on the flow cytometer. A gate was used to eliminate debris and then histograms were plotted for each flow cytometry channel needed in the immunophenotyping panels. The histograms represent normalized cell counts and their fluorescence intensity in each channel. The plots are representative of one out of three replicates run for each condition from Donor I3L4.

Before conducting assay performance qualification with given nanomaterials, it is important to test the compatibility of nanomaterials with the NovoCyte 3005 because some nanomaterials have intrinsic fluorescence that has the potential to cause optical interferences with the flow cytometer settings which would eliminate or decrease the ability of immunophenotyping panels to be used to characterize such particles. Therefore, we used Doxil to determine if it interfered with the lasers, filters and detector sets used in the immunophenotyping panels described above. Doxil was chosen to test for interference because of doxorubicin's inherent fluorescence caused by the anthracycline chromophore group which is known to interfere with the red and green channels of cytometers (40–42). Healthy donor PBMC from one donor were treated with varying concentrations of Doxil (0.2033, 0.0203, 0.0041, or 0.0008 mg/ml) overnight and then run on the NovoCyte 3005. A primary gate was drawn to eliminate debris and then the cells were plotted with each channel on the x-axis (Figure 3). It was determined that the highest concentration of Doxil (0.2033 mg/ml) interferes with many of the channels including FITC, PE, PE-Cy7, BV5710, BV650, BV711 and BV785; however, at the lowest two concentrations of Doxil used (0.0041 and 0.0008 mg/ml), interferences were essentially eliminated (Figure 3). Therefore, these immunophenotyping panels could only be used to determine immune cell effects of Doxil if the lower concentrations are relevant to the study objectives. An alternative to evaluate higher Doxil concentrations is to avoid using the affected channels and fluorophores and select other channels or fluorophores when available.

### 3.5. Effect of dendrimers on immune cell populations

**Figure 4:**
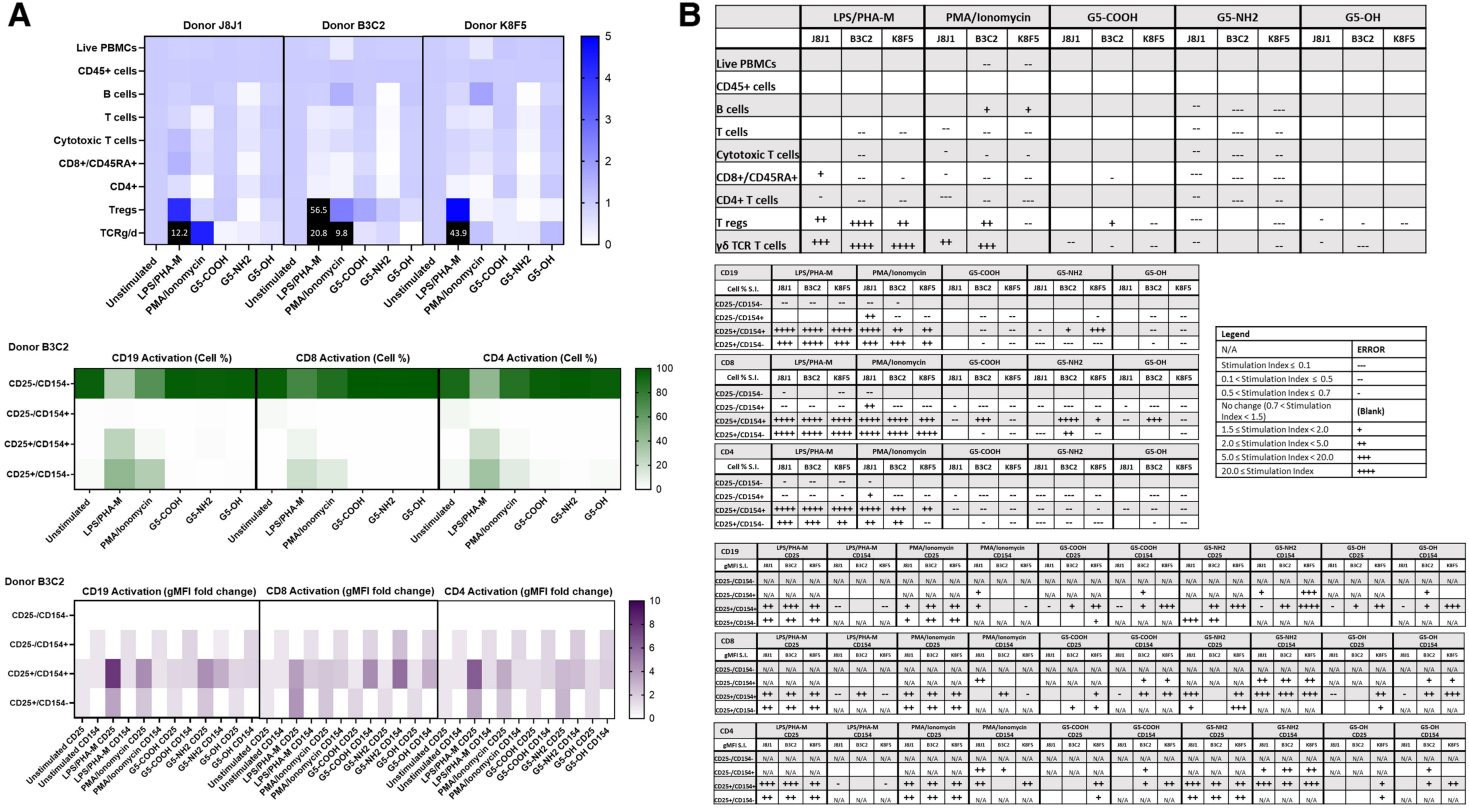
G5-NH2 dendrimers modify cell population and activation statuses in immunophenotyping panel #1. PBMC were treated with positive controls (LPS/PHA-M or PMA/Ionomycin), 10 µg/ml G5 dendrimers (G5-COOH, G5-NH2, or G5-OH), or left unstimulated/untreated for 24 h. (**A**) The top heat map indicates the average stimulation index (SI) of percentage of treated cell populations as compared to unstimulated/untreated populations for each of the three heathy donors—each donor had two replicates for each condition. Cell population percentages were defined as percentage of live cells. The middle heat map is a representative heat map of the average percentage of cells in each of the different activation quadrants for CD19, CD8, and CD4 cells from Donor B3C2 (2 replicates per condition). The bottom heat map is a representative heat map from Donor B3C2 (2 replicates per condition) indicating the average stimulation index of the geometric mean fluorescence intensity (gMFI) of the activation markers in each cell population as compared to the unstimulated cells. (**B**) Tables representing the average stimulation indices of the different cell populations as compared to the unstimulated PBMC for each of the three donors (2 replicates per condition for each of the 3 donors). The top table indicates changes in cell populations. The next three tables indicate fold changes in percentages of activated cells in CD19, CD8, and CD4 populations and the last three indicate the fold changes for gMFI of activated cells in CD19, CD8, and CD4 populations.

**Figure 5:**
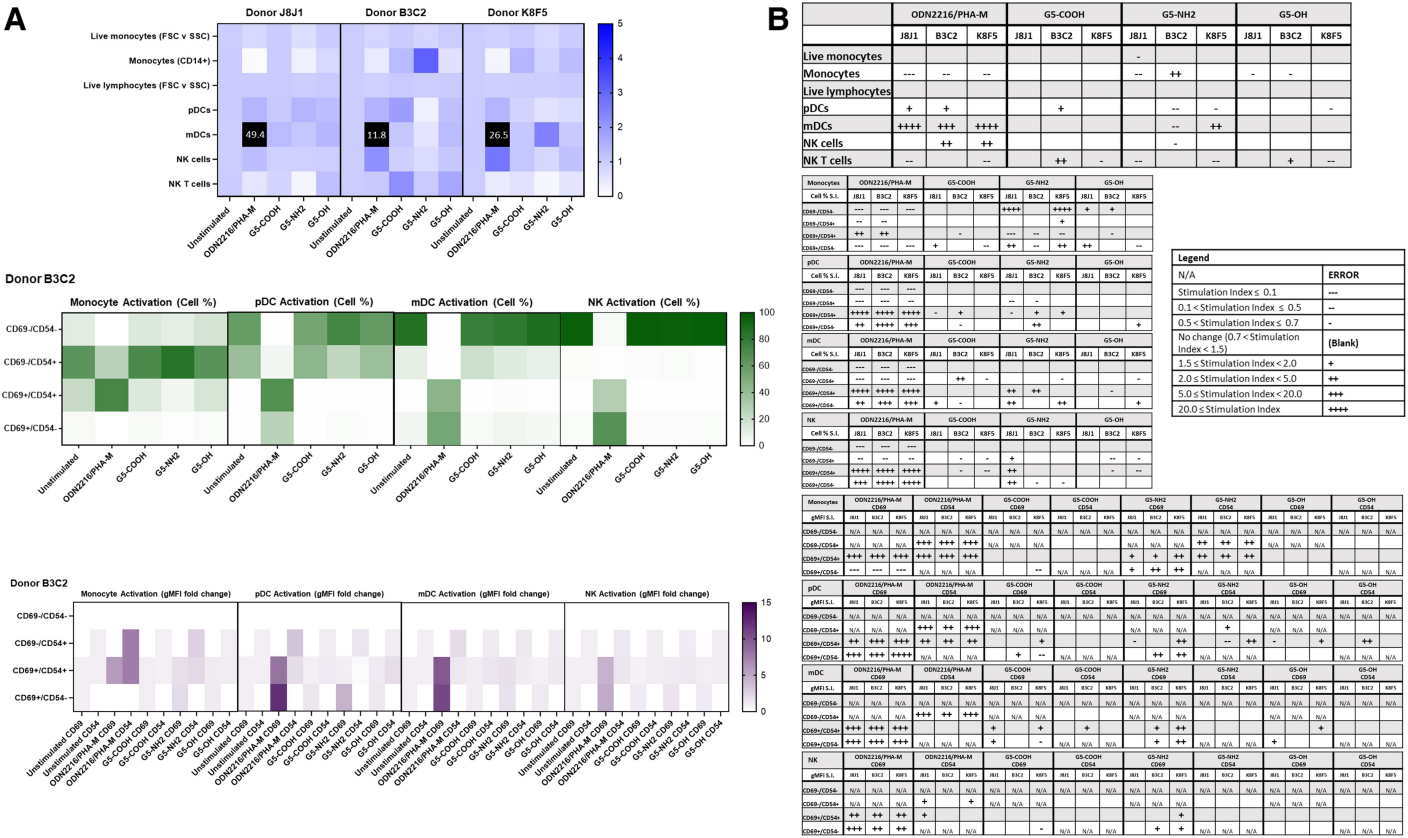
G5-NH2 dendrimers modify cell population and activation statuses in immunophenotyping panel #2. PBMC were treated with positive controls (ODN2216/PHA-M), 10 µg/ml G5 dendrimers (G5-COOH, G5-NH2, or G5-OH), or left unstimulated/untreated for 24 h. (**A**) The top heat map indicates the average stimulation index (SI) of percentage of treated cell populations as compared to unstimulated (or untreated) populations for each of the three heathy donors—each donor had two replicates for each condition. Cell population percentages were defined as percentage of live cells. The middle heat map is a representative heat map of the average percentage of cells in each of the different activation quadrants for monocyte, pDC, mDC and NK cells from Donor B3C2 (2 replicates per condition). The bottom heat map is a representative heat map from Donor B3C2 (2 replicates per condition) indicating the average stimulation index of the geometric mean fluorescence intensity (gMFI) of the activation markers in each cell population as compared to the unstimulated cells. (**B**) Tables representing the stimulation indices of the different cell populations as compared to the unstimulated PBMC for each of the three donors (2 replicates per condition for each of the 3 donors). The top table indicates changes in cell populations. The next four tables indicate fold changes in percentages of activated cells in monocytes, pDC, mDC and NK cell populations and the last four tables indicate the fold changes for gMFI of activated cells in monocyte, pDC, mDC and NK cell populations.

Commercially available research-grade nanomaterials without intrinsic fluorescent properties were then used for assay performance qualification. G5 dendrimers with various physicochemical characteristics were used—including G5-COOH, G5-OH, and G5-NH2 dendrimers. PCC (DLS and zeta potential) and endotoxin limits of the dendrimers were assessed prior to flow cytometry (Supplementary Figure S6 and Table S1). These dendrimers are expected to have varying effects on immune cell populations given their known physicochemical properties. These dendrimers at 10 µg/ml were analyzed using both immunophenotyping panels in three healthy donor PBMC. Among the generation 5 dendrimers with three surface functionalities—amine (cationic), carboxy (anionic) and hydroxy (neutral)—G5-NH2 dendrimers resulted in the most pronounced effects on the immune cell populations in the Immunophenotyping Panel #1; in most cases, they decreased the cell population percentages as compared to unstimulated controls (Figure 4). The activation of immune cell subsets was also assessed both in terms of changes in the number of cells in each activation quadrant and the geometric fluorescence intensity (gMFI) of cells in each quadrant. Across the three donors, 10 µg/ml G5 dendrimers did not significantly increase the percentage of cells in the activated quadrants; however, G5-NH2 dendrimers more consistently increased the gMFI of the activated cell populations (Figure 4B). Immunophenotyping Panel #2 data revealed again that G5-NH2 dendrimers caused the greatest changes in cell populations (Figure 5). G5-NH2 dendrimers increased gMFI of CD69 and CD54 activation markers in monocytes across all donors but caused inter-donor variation for cell percentage activation and gMFI in other analyzed populations.

**Figure 6:**
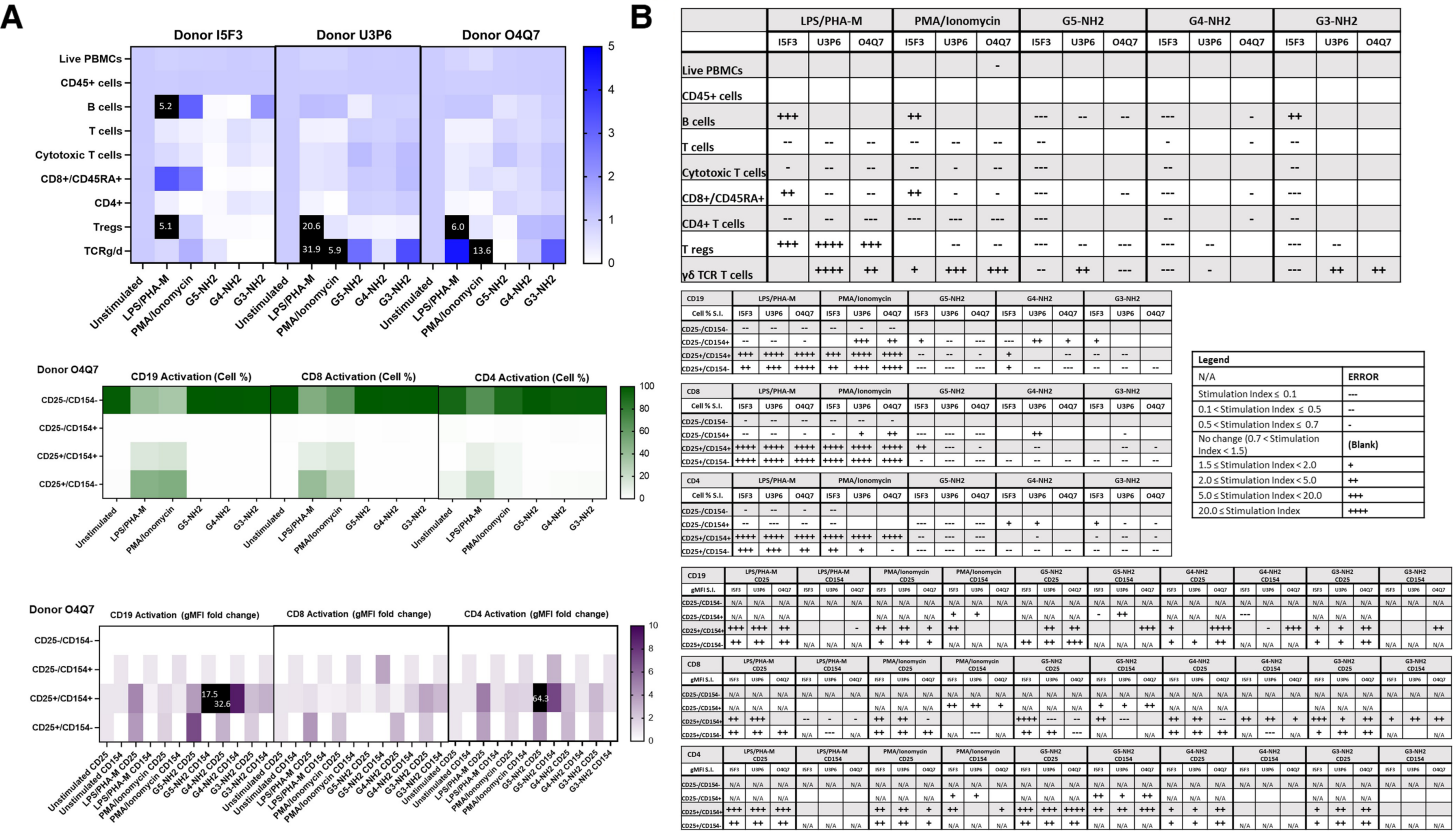
NH2-terminated dendrimers modify cell population and activation statuses in immunophenotyping panel #1. PBMC were treated with positive controls (LPS/PHA-M or PMA/Ionomycin), 10 µg/ml NH2-terminated dendrimers (G5-NH2, G4-NH2, or G3-NH2), or left unstimulated/untreated for 24 h. (**A**) The top heat map indicates the average stimulation index (SI) of percentage of treated cell populations as compared to unstimulated (or untreated) populations for each of the three human donors—each donor had two replicates for each condition. Cell population percentages were defined as percentage of live cells. The middle heat map is a representative heat map of the average percentage of cells in each of the different activation quadrants for CD19, CD8, and CD4 cells from Donor O4Q7 (2 replicates per condition). The bottom heat map is a representative heat map from Donor O4Q7 (2 replicates per condition) indicating the average stimulation index of the geometric mean fluorescence intensity (gMFI) of the activation markers in each cell population as compared to the unstimulated cells. (**B**) Tables representing the stimulation indices of the different cell populations as compared to the unstimulated PBMC for each of the three donors (2 replicates per condition for each of the 3 donors). The top table indicates changes in cell populations. The next three tables indicate fold changes in percentages of activated cells in CD19, CD8, and CD4 populations and the last three indicate the fold changes for gMFI of activated cells in CD19, CD8, and CD4 populations.

**Figure 7:**
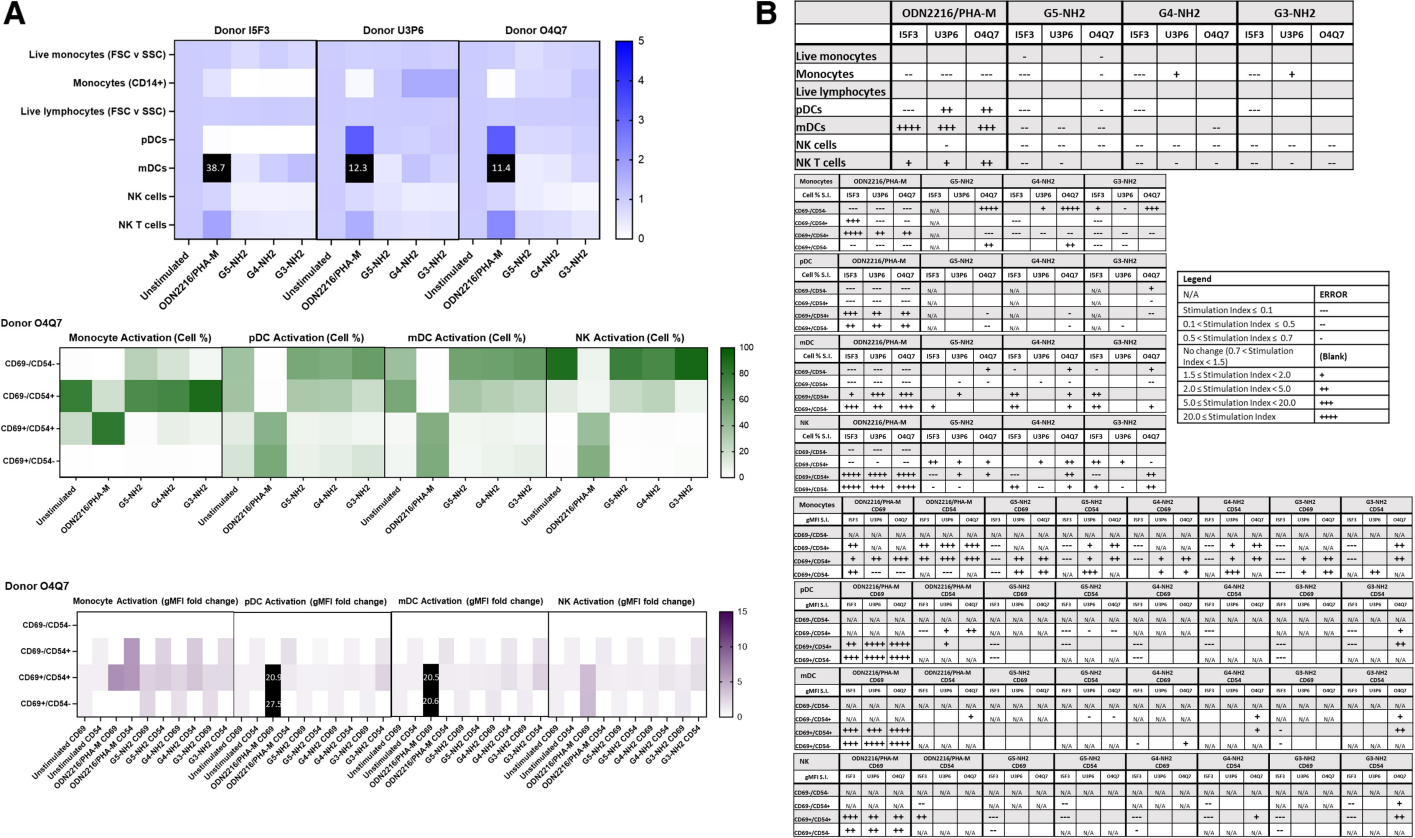
NH2-terminated dendrimers modify cell population and activation statuses in immunophenotyping panel #2. PBMC were treated with positive controls (ODN2216/PHA-M), 10 µg/ml NH2-terminated dendrimers (G5-NH2, G4-NH2, or G3-NH2), or left unstimulated/untreated for 24 h. (**A**) The top heat map indicates the average stimulation index (SI) of percentage of treated cell populations as compared to unstimulated (or untreated) populations for each of the three human donors—each donor had two replicates for each condition. Cell population percentages were defined as percentage of live cells. The middle heat map is a representative heat map of the average percentage of cells in each of the different activation quadrants for monocyte, pDC, mDC and NK cells from Donor O4Q7 (2 replicates per condition). The bottom heat map is a representative heat map from Donor O4Q7 (2 replicates per condition) indicating the average stimulation index of the geometric mean fluorescence intensity (gMFI) of the activation markers in each cell population as compared to the unstimulated cells. (**B**) Tables representing the stimulation indices of the different cell populations as compared to the unstimulated PBMC for each of the three donors (2 replicates per condition for each of the 3 donors). The top table indicates changes in cell populations. The next four tables indicate fold changes in percentages of activated cells in monocyte, pDC, mDC and NK cell populations and the last four tables indicate the fold changes for gMFI of activated cells in monocyte, pDC, mDC and NK cell populations.

Because the amine, but not carboxy or hydroxy-terminated, G5 dendrimers caused the greatest variations in cell populations and activation status, we chose amine-terminated dendrimers of various generations for a subsequent experiment. G5, G4, and G3 amine-terminated dendrimers (G5-NH2, G4-NH2 and G3-NH2) at concentration 10 µg/ml were compared to assess the role of nanoparticle size in dendrimer-mediated changes of immune cell populations. It was determined that in three healthy donors, various generations of NH2-surface dendrimers generally had similar effects on cell population and activation status in both Immunophenotyping Panel #1 (Figure 6) and Immunophenotyping Panel #2 (Figure 7). The variations of changes in cell populations and activation statuses caused by the different generations of NH2-terminated dendrimers were less distinct than the differences between the effects caused by the G5 dendrimers with different surface functionalities (amine, carboxy and hydroxy).

### 3.6. Effect of AmBisome and Feraheme on immune cell populations

**Figure 8:**
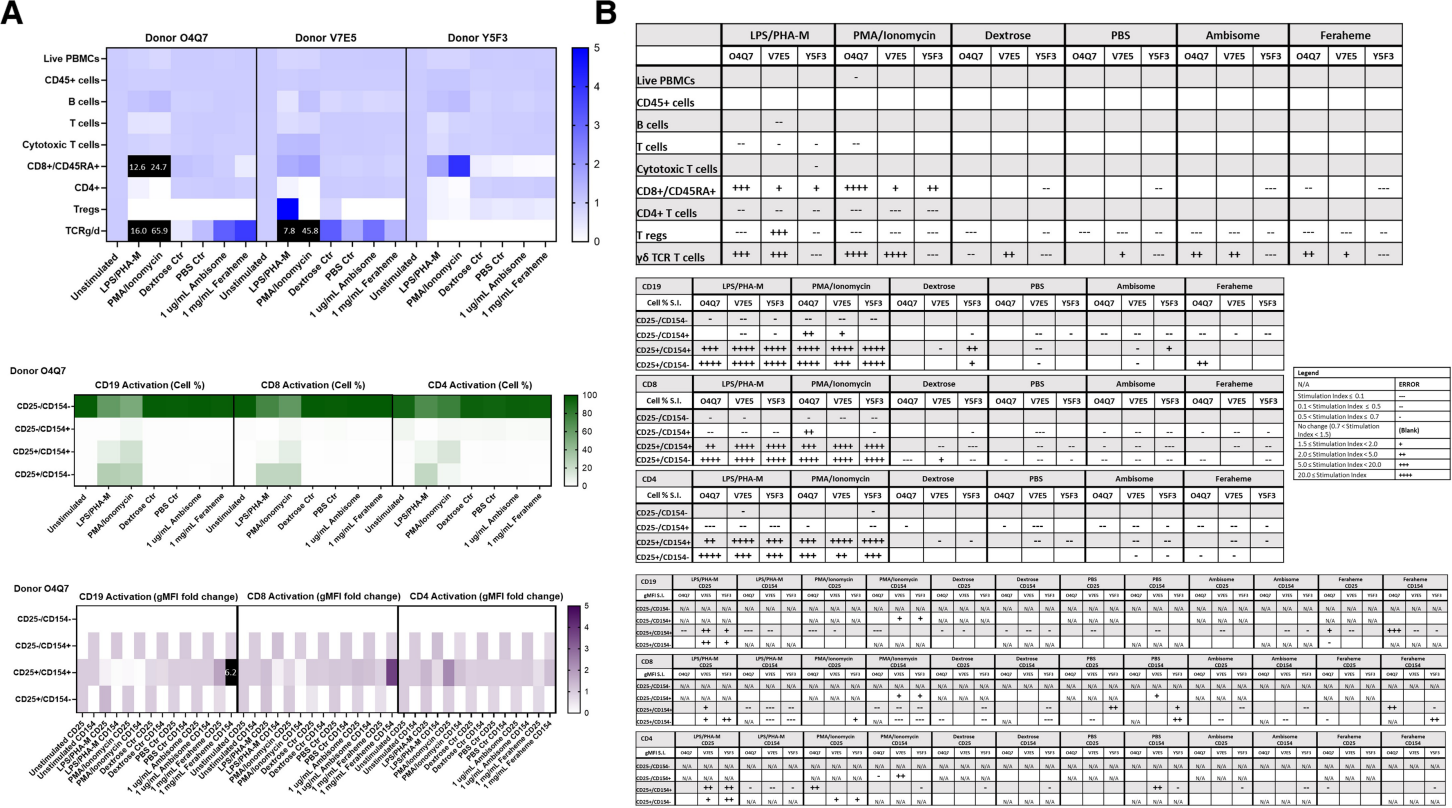
AmBisome nor Feraheme modify cell population and activation statuses in immunophenotyping panel #1. PBMC were treated with positive controls (LPS/PHA-M or PMA-Ionomycin), negative controls (unstimulated/untreated, Dextrose Control, PBS Control), 1 µg/ml AmBisome or 1 mg/ml Feraheme for 24 h. Dextrose was a control for AmBisome and PBS was a control for Feraheme. (**A**) The top heat map indicates the average stimulation index (SI) of percentage of treated cell populations (AmBisome or Feraheme) as compared to unstimulated (or untreated) populations for each of the three human donors—each donor had two replicates for each condition. Cell population percentages were defined as percentage of live cells. The middle heat map is a representative heat map of the average percentage of cells in each of the different activation quadrants for CD19, CD8, and CD4 cells from Donor O4Q7 (2 replicates per condition). The bottom heat map is a representative heat map from Donor O4Q7 (2 replicates per condition) indicating the average stimulation index of the geometric mean fluorescence intensity (gMFI) of the activation markers in each cell population as compared to the unstimulated cells. (**B**) Tables representing the average stimulation indices of the different cell populations as compared to the unstimulated PBMC for each of the three donors (2 replicates per condition for each of the 3 donors). The top table indicates changes in cell populations. The next three tables indicate fold changes in percentages of activated cells in CD19, CD8, and CD4 populations and the last three indicate the fold changes for gMFI of activated cells in CD19, CD8, and CD4 populations.

**Figure 9:**
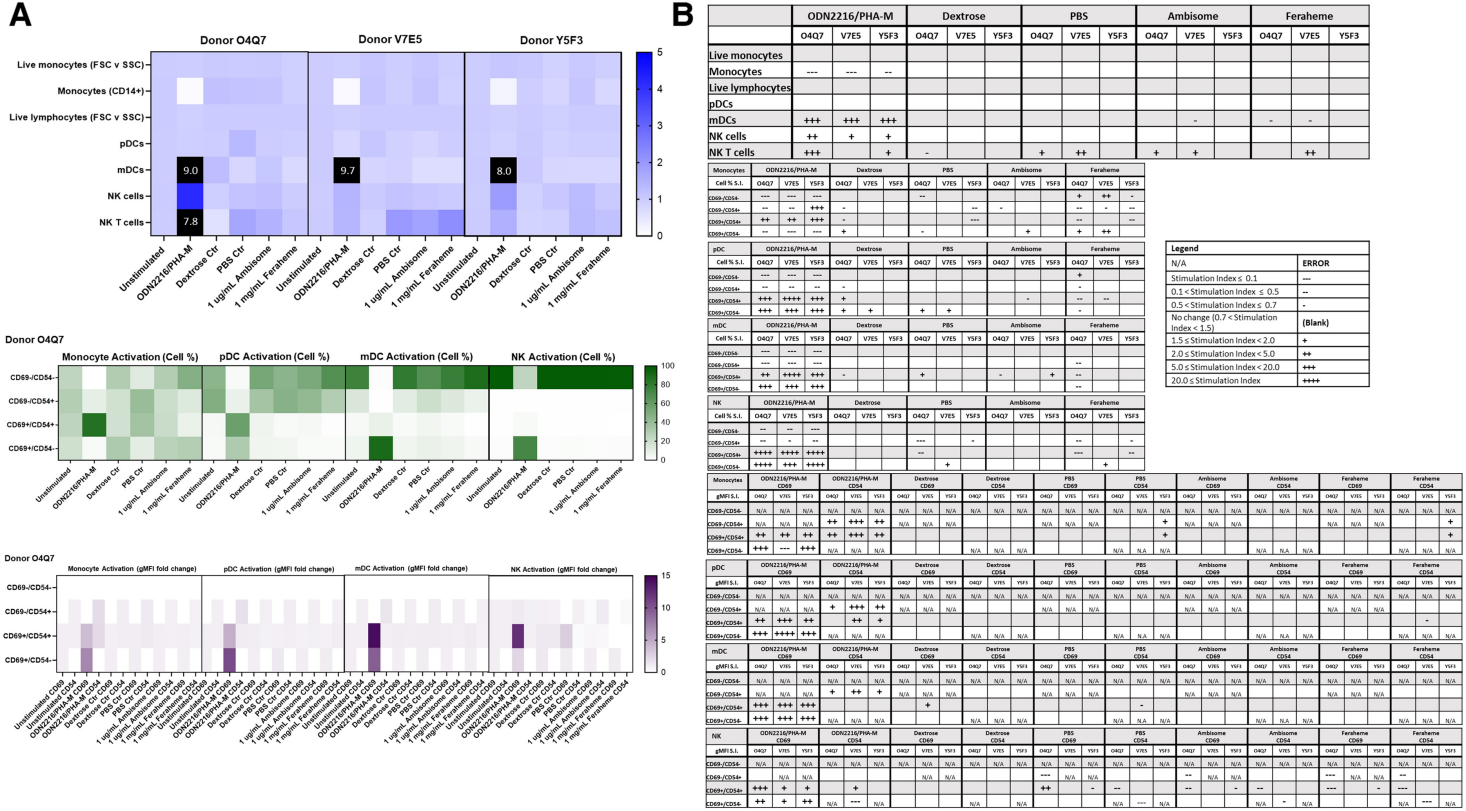
AmBisome nor Feraheme modify cell population and activation statuses in immunophenotyping panel #2. PBMC were treated with positive controls (LPS/PHA-M or PMA/Ionomycin), negative controls (unstimulated/untreated, Dextrose Control, PBS Control), 1 µg/ml AmBisome or 1 mg/ml Feraheme for 24 h. Dextrose was a control for AmBisome and PBS was a control for Feraheme. (**A**) The top heat map indicates the stimulation index (SI) of percentage of treated cell populations (AmBisome or Feraheme) as compared to unstimulated (or untreated) populations for each of the three human donors—each donor had two replicates for each condition. Cell population percentages were defined as percentage of live cells. The middle heat map is a representative heat map of the average percentage of cells in each of the different activation quadrants for monocyte, pDC, mDC and NK cells from Donor O4Q7(2 replicates per condition). The bottom heat map is a representative heat map from Donor O4Q7 (2 replicates per condition) indicating the average stimulation index of the geometric mean fluorescence intensity (gMFI) of the activation markers in each cell population as compared to the unstimulated cells. (**B**) Tables representing the average stimulation indices of the different cell populations as compared to the unstimulated PBMC for each of the three donors (2 replicates per condition for each of the 3 donors). The top table indicates changes in cell populations. The next four tables indicate fold changes in percentages of activated cells in monocyte, pDC, mDC and NK cell populations and the last four tables indicate the fold changes for gMFI of activated cells in monocyte, pDC, mDC and NK cell populations.

Commercially available clinical nanomaterials were then used for assay performance qualification on the immunophenotyping panels. AmBisome, a clinical liposome containing amphotericin B, and Feraheme, an iron oxide, were both used to validate the panels. Immunophenotyping Panels #1 and #2 were both assessed in three healthy donors. Vehicle controls, 5% Dextrose Injection and 1× PBS, were used for AmBisome and Feraheme controls, respectively. The cell populations and their activation statuses for Immunophenotyping Panel #1 were assessed and it was determined that neither 1 µg/ml AmBisome nor 1 mg/ml Feraheme elicited consistent changes in cell population or activation statuses (CD25 and CD154) across the three donors (Figure 8). Similarly, Immunophenotyping Panel #2 results indicated that neither 1 µg/ml AmBisome nor 1 mg/ml Feraheme impacted the monocyte, DC, or NK cell populations and activations statuses across all three donors (Figure 9).

### 3.7. Applying statistical algorithms to analyze immunophenotyping data

**Figure 10:**
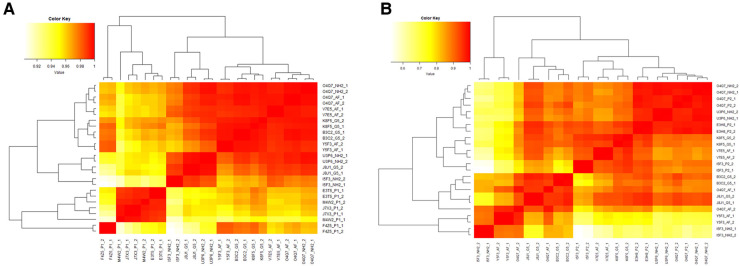
Heat map of Pearson's R values for immunophenotyping panels. The cell type percentage values of the negative controls were compared between repeat pairs (technical replicates) and even within donors (donors repeated across conditions) for Immunophenotyping Panel #1 (**A**) and Immunophenotyping Panel #2 (**B**) to determine correlation coefficients and the reproducibility of the panels.

To assess the reproducibility of the immunophenotyping results, an initial statistical analysis of unstimulated (negative controls) was performed. Cell type percentages of unstimulated (negative) controls from all experimental runs were compared using Pearson's correlation. For Immunophenotyping Panel #1, there is high correlation between repeat pairs (technical replicates) and even within donors (donors repeated across conditions) when considering all cell type percentages (Figure 10A). There is also evidence of three donor subgroups in the data. For Immunophenotyping Panel #2, when considering all cell type percentages there is a similar trend as seen in Immunophenotyping Panel #1 and good agreement in the repeated pairs (Figure 10B). For this panel, donor O4Q7 showed relatively high correlation between conditions while donor I5F3 did not (Figure 10B).

Statistical analysis regarding the treatment conditions was then performed. Student's paired *t*-test and one-way ANOVA are statistical algorithms commonly used in biomedical research to get an insight into the significance of differences observed between two or multiple samples, respectively. A *t*-test was not initially selected for this study due to the nature of the resulting data sets (i.e., non-normal, highly correlated, proportional), so we experimented with non-parametric tests (e.g., rank-based), count-based tests, and linear models. However, with an N of 3, the loss of statistical power we experienced in using these alternative methods made finding any significant results impossible (e.g., the minimum *p*-value obtainable for a two-sided Wilcoxon signed rank test with an *N* = 3 is 0.25). A large number of multiple comparisons further complicated the analysis. Any traditional *p*-value correction would eliminate many, if not all, of our findings. Additionally, correction for multiple hypotheses generally assumes the data is independent. Here, however, the data is very dependent as one activation or cell type percentage directly affects several others. This made determining the exact family-wise degrees of freedom not feasible.

**Figure 11:**
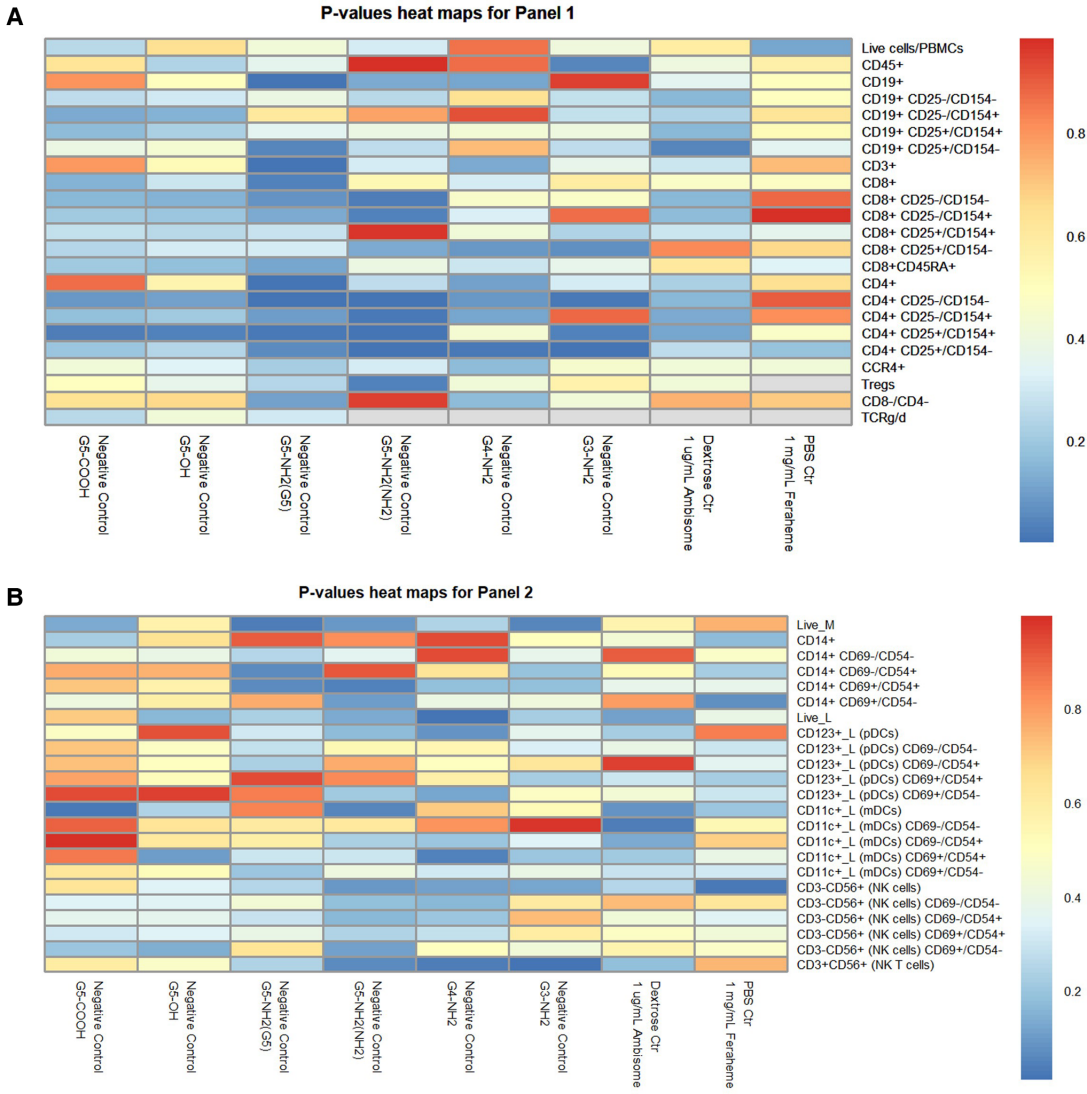
Heat map of *p*-values for immunophenotyping panels. The average percentage values of each cell type or activation quadrant of negative control (or vehicle control) were compared to the treatment group via *t*-tests with permutation analyses for Immunophenotyping Panel #1 (**A**) and Immunophenotyping Panel #2 (**B**). (**A**) For Immunophenotyping Panel #1, any real *p*-value below 0.1230239 is equivalent to permutated *p*-value <0.05 and is the most significant. (**B**) For Immunophenotyping Panel #2, any real *p*-value below 0.3320055 is equivalent to permutated *p*-value <0.05 and is the most significant.

To overcome these limitations, we performed the permutation analysis across all of the data using a previously described approach (37). For each *t*-test, we shuffled the treatment versus control labels for the donor pairs (keeping the data pairing and all cell activation ratio structure of the data intact). Due to the sample size of three, there were only three unique permutations available for each *t*-test (i.e., with two-sided statistical tests, switching the treatment/control labels for the first of three donors is equivalent to switching the other two instead). This permutation of the data gave us three permuted *p*-values for each real one. This distribution of permuted *p*-values provides a null hypothesis distribution against which to compare our real *p*-values. If a real *p*-value for a given cell type and sample is lower than most or all the permuted ones, we have confidence that it was unlikely to have been found by random chance. The formula for this is (number of permuted *p*-values lower than our real one +1)/(total number of permuted *p*-values +1) as described earlier (37). For this permutation analysis, we ignored any *t*-test where the sample size dropped below 3 due to missing data. For Immunophenotyping Panel #1, any real *p*-value below 0.1230239 would be in the top 5% most significant of the permuted ones (i.e., it would have a permutation *p*-value of <0.05) (Figure 11A). The “value to beat” for Immunophenotyping Panel #2 is likewise 0.3320055 (Figure 11B). The mean difference and *p*-values for Immunophenotyping Panel #1 are presented in Supplementary Table S2, respectively; that for Immunophenotyping Panel #2 are shown in Supplementary Table S3.

## 4. Discussion

There is a lack of standardized methods for immunophenotyping in the preclinical nanomedicine research setting. To the best of our knowledge, immunophenotyping panels specifically available for the NovoCyte 3005 assessing the lymphoid and myeloid-derived cell population discussed here within have not been previously developed. Therefore, the two distinct panels developed in our laboratory can aid in assessing immunological impact of various nanomaterials. These two panels were developed to focus on some of the most abundant PBMC populations that have been shown to contribute to nanoparticle-mediated immune responses while adhering to the limitations of the NovoCyte 3005. Additional immunophenotyping panels that either include markers to identify various granulocytes or dive deeper into different lymphoid cell populations, such as different T cell memory population or other innate-like T cells, could be developed for future studies. Furthermore, in this study practical limitations—volume of nanoparticles required per assay per donor and the number of test sample needed to accommodate necessary controls—restricted our assay validation donor number to *N* = 3–4. This methodology is helpful to identify trends in nanoparticle effects on various populations of the blood cells with the aim of prioritizing other immunotoxicity studies. In order to increase statistical power of the findings and for results to be more quantitative in nature, a higher number of donors should be used. In general, assay performance qualification is based on 3–4 donors; this number increases to 10 for assay validation and to 50–100 for the full study (43). Since early preclinical development of nanomaterials is often associated with limited amounts of study samples and funding, robust methodologies and decision trees that may help researchers to prioritize limited resources are highly needed. The methodology presented herein, when used in conjunction with a decision tree (Supplementary Figure S7), represents one such approach which may help researchers prioritize assays for immunotoxicity studies.

Our antibody titration experiments revealed that the company-provided recommended dilutions for each antibody were far higher than the optimal concentrations and, therefore, will save considerable amounts of reagents per experiment (Supplementary Figure S1). Single stain controls and FMO controls for both panels showed that proper compensation could be determined. Although the compensation for some of the antibodies was high, the ability to compensate the panels remained unhindered (Supplementary Figures S2–S5).

Furthermore, it was determined that the immunophenotyping panels developed here within could indeed detect the defined cell populations (Tables 3, 4) and could determine increases in activation of cells stimulated with positive controls (Figures 1, 2). The technical replicates and repeated donors also revealed that the panels were stable and reproducible—with Immunophenotyping Panel #1 showing better reproducibility than Immunophenotyping Panel #2 (Figure 10). This could be explained by the nature of cell populations analyzed in each panel; particularly, Panel #1 targets cells involved in the adaptive immune response which are expected to vary between individual donors but be less sensitive to the *in vitro* administered treatments due to the limits of *in vitro* systems to the adaptive immune response and short (24 h) time of *in vitro* treatment. Unlike Panel #1, cell populations targeted by the Panel #2 are expected to be more sensitive to *in vitro* manipulations due to their involvement in the innate immune response which is also expected to vary widely between individual donors. Despite the reproducibility, however, some (less abundant in PBMC) cell populations such as naïve (CD45RA^+^) CD8^+^ T cells, *T*_regs_, γδ T cells, and NKT cells have highly variable and/or small counts and should be considered carefully.

It is also important to recognize that the presence of these and other cell populations could also change with different donor populations (e.g., healthy vs. patient) and be further influenced by a condition (e.g., immunosuppression vs. hypersensitivity). Therefore, we suggest that the assay performance qualification runs are performed for each donor population to identify the influence of these conditions on assay performance and make adjustments, if needed.

The procedure one follows to stain, wash, fix the cells should also be noted as variations in staining buffer have been seen to cause differences in quantification of cell populations (data not shown). Furthermore, the statistical analysis of the impact of various nanomaterials on cell makeup and activity was complicated by low sample number, high dependency between conditions, and large number of multiple comparisons. Therefore, the immunological data gained from such experimentation should be considered on a more qualitative level rather than quantitatively. We propose using these immunophenotyping panels to identify cell populations impacted by the nanomaterial of interest and then proceed with a more detailed analysis of these population using available cells line model (if available) or enriched primary cell populations isolated via negative or positive selection using commercially available kits and reagents. We further recommend applying *in vitro* and *in vivo* immune function test to verify the initial findings these studies.

The immunophenotyping panels assessing both research and clinical grade nanomaterials confirmed that their immunological impact can be reliably determined and agree with previously established research. However, the nanomaterials that are assessed by these immunophenotyping panels must be free of certain inherent properties that interfere with the NovoCyte 3005. Interference of nanoparticles can be determined prior to performing full immunophenotyping assessment. For example, the Doxil formulation used in our study interfered with the accurate method performance due to the known fluorescent properties of its active pharmaceutical ingredient—doxorubicin—containing the anthracycline chromophore group with previously described interference with the red and green channels of flow cytometers (40–42). This interference was overcome by the sample dilution (Figure 3); therefore, dilution of the interfering samples may be used to overcome this barrier as long as the resulting non-interfering concentrations remain relevant to the study objectives. If the analysis of Doxil or other interfering formulations requires the use of higher concentrations, alternative methods should be considered to generate results that would provide relevant to the study objectives information. One such alternative is mass cytometry (CyTOF) employing heavy metals for antibody labeling with the subsequent detection of these antibodies by a mass spectrometer (44–46).

Analysis of dendrimers in our study allowed us to both evaluate the method performance for nanomaterials with different compositions and gain an insight into the structure-activity relationship (SAR). Earlier SAR studies revealed that large PAMAM dendrimers with amine-surface functionality are the most reactive with immune cells (reviewed in (47)). Therefore, we employed them for the assay performance qualification. Among generation 5 dendrimers, cationic (G5-NH2) dendrimers had greater influence on the number and the activation status of immune cells than their anionic (G5-COOH) and neutral (G5-OH) counterparts influenced (Figures 4, 5, 11). This finding agrees with our earlier studies in platelets and leukocytes (48–50). Since tested dendrimers contained trace amounts of endotoxin (Supplementary Table S1), we hypothesize that the observed effects on the activation status of immune cells are likely due to the immunomodulation, a phenomenon in which dendrimers *per se* and a low concentration of endotoxin alone do not result in a detectable activation of immune cells but a combination of these, otherwise ineffective, treatments result in detectable effect (51). A control sample of endotoxin at a concentration equivalent to that found in dendrimers was included in the analysis and as expected, did not produce any detectable changes in the cell number and activation status (data not shown).

To gain an insight into the role of nanoparticle size, we next tested amine-terminated dendrimers of different generations. Unlike our earlier studies in platelets and leukocytes demonstrating clear size-dependent effects changing from generation to generation proportionally to the change in the number of surface amines (49, 50), when used in the current immunophenotyping study, G3-NH2, G4-NH2 and G5-NH2 dendrimers did not result in a clear generation-dependent effects and induced comparable changes in cell populations (Figures 6, 7). This is not surprising, provided the complexity of interactions between various subsets of immune cells present in PBMC samples and the 24 h incubation time during which the initial direct size-dependent effects of particles on a particular subset of immune cells are neutralized by both secondary messengers acting via autocrine and/or paracrine loops. This finding also points to the utility of this assay for qualitative rather than quantitative analysis of nanoparticle effects on the immune cell subsets.

At tested concentrations, two other clinical-grade formulations—liposomal amphotericin (AmBisome) and iron oxide (Feraheme)—did not result in consistent changes in cell population and or activation (Figures 8, 9, 11). Considering these nanomaterials are used in the clinic, the lack of effect on immune cell populations was not surprising. Earlier studies in our laboratory demonstrated that pre-exposure of primary human T-cells to Feraheme results in subsequent suppression of their function in response to activating stimuli such as CD3 antibody; this property was helpful in alleviating the T-cell mediated inflammation *in vivo* (30, 52). Similar observations were also reported by others for the iron-oxide-based imaging agent Resovist both *in vitro* and *in vivo* (53–55). Our current study design aimed at identifying the direct effects of nanoparticles on the number and activation status of lymphoid and myeloid cells present in PBMC and did not include a treatment arm to study potential immunosuppressive or immunomodulatory effects. However, we propose that the same protocol and panels can be used to analyze how pre-exposure to nanoparticles would change the activation status of tested lymphoid and myeloid cells in response to the respective assay positive controls.

Overall, this study demonstrated that after some site-specific method optimization these two distinct immunophenotyping panels could be used by research laboratories to assess immunological impact of nanomaterials and thus contribute to the SAR studies and inform nanoparticle efficacy and safety evaluation. The impact of nanomaterials on individual cell subsets identified using the immunophenotyping protocol described herein bears a qualitative character, requires further verification using enriched cell populations and immune function tests, and, therefore, should be considered as an initial screening step of nanomaterials immunocompatibility. Finally, Pearson's correlation analysis is useful in determining the assay reproducibility based on the positive and negative controls, whereas the permutation analysis described by Phipson and Smyth (37) is more helpful in identifying the cell subsets affected by test nanomaterials.

## Data Availability

The original contributions presented in the study are included in the article/**Supplementary Material**, further inquiries can be directed to the corresponding author.
