# Supplementary material for: Multicolor flow cytometry-based immunophenotyping for preclinical characterization of nanotechnology-based formulations: an insight into structure activity relationship and nanoparticle biocompatibility profiles
Source: Front Allergy. 2023 Jul 4;4:1126012. doi: 10.3389/falgy.2023.1126012 (PMC10353541; doi:10.3389/falgy.2023.1126012)
Supplement: Supplementary file 1 [file Table1.docx]

Supplementary Material

Multicolor flow cytometry-based immunophenotyping for preclinical characterization of nanotechnology-based formulations: an insight into structure activity relationship and nanoparticle biocompatibility profiles

Hannah S. Newton^1^, Jenny Zhang^2^, Duncan Donohue^3^, Ragi Unnithan^3^, Edward Cedrone^1^, Jie Xu^1^, Alison Vermilya^1^, Tyler Malys^3^, Jeffrey D. Clogston^1^, Marina A. Dobrovolskaia^1,*^

^1^ Nanotechnology Characterization Laboratory, Cancer Research Technology Program, Frederick National Laboratory for Cancer Research Sponsored by the National Cancer Institute, Frederick, MD 21702, USA.

^2^ Agilent Technologies, Santa Clara, CA 95051 USA.

^3^ Statistics Department, Data Management Services Inc., Frederick National Laboratory for Cancer Research Sponsored by the National Cancer Institute, Frederick, MD 21702, USA.

*** Correspondence:**Marina Dobrovolskaia
[marina@mail.nih.gov](mailto:marina@mail.nih.gov)


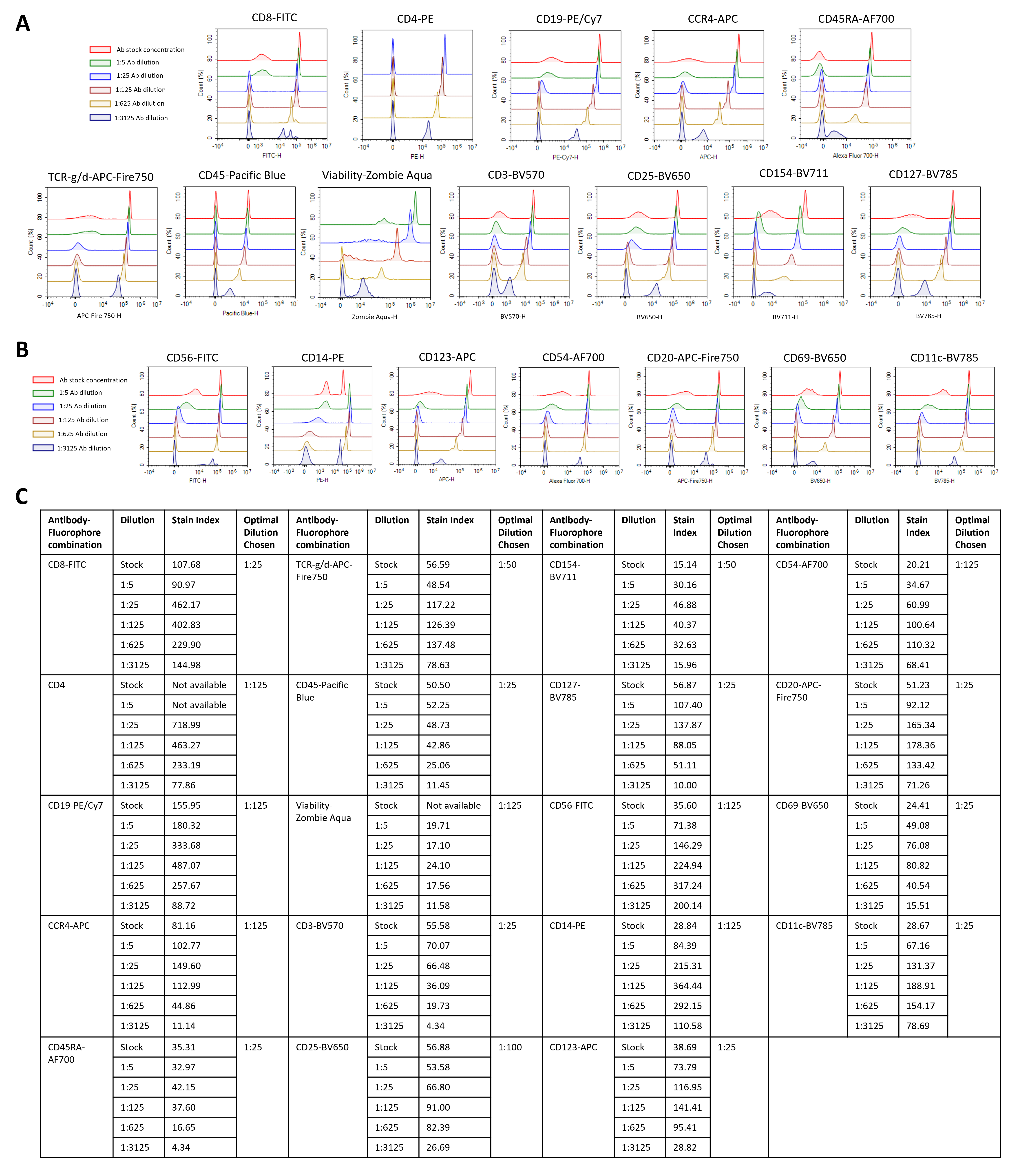


**Figure S1. Antibody titrations for Immunophenotyping Panels.** **(A)** Compensation beads stained with serial dilutions of a single antibody (or dye) for each antibody used in Immunophenotyping Panel #1 and those with overlapping use for Immunophenotyping Panel #2. **(B)** Compensation beads stained with serial dilutions of a single antibody for each antibody used in Immunophenotyping Panel #2. **(C)** A table specifying the numerical evaluation of antibody titrations for the antibody-fluorophore combinations indicated in (A) and (B). This table lists each antibody-fluorophore used, dilutions measured, calculated stain index via NovoExpress, and the optimal dilution chosen.


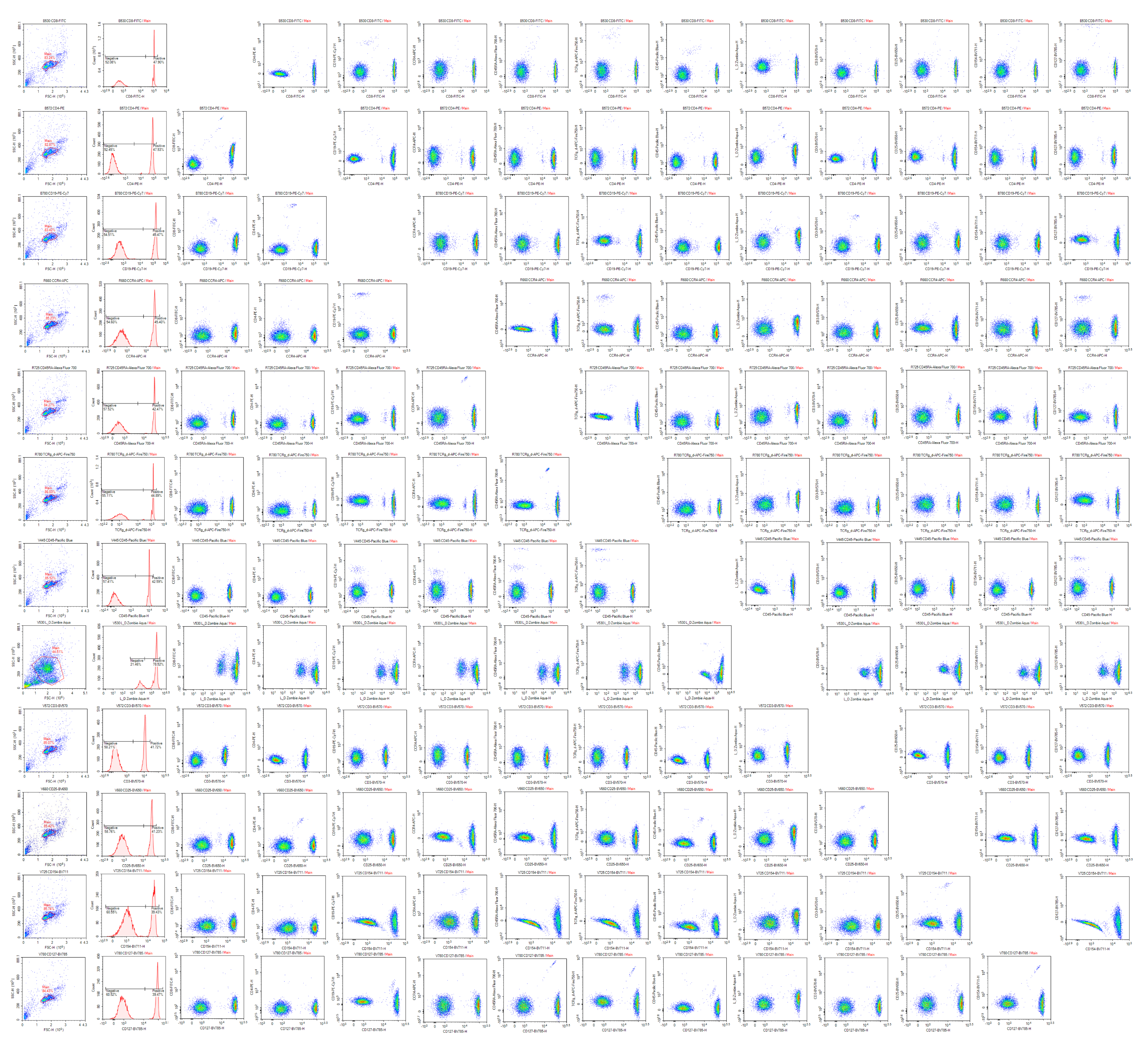


**Figure S2. Representative Immunophenotyping Panel #1 single stain compensation.** A representative replicate of Immunophenotyping Panel #1 single stain controls run with compensation beads and PBMC. Each row of plots is a different single stain control. The first dot plot selects the main population of beads or PBMC. The second plot (histogram) identifies the negative and positive events. The remaining plots in each row show the negative and positive events plotted against each different channel used.


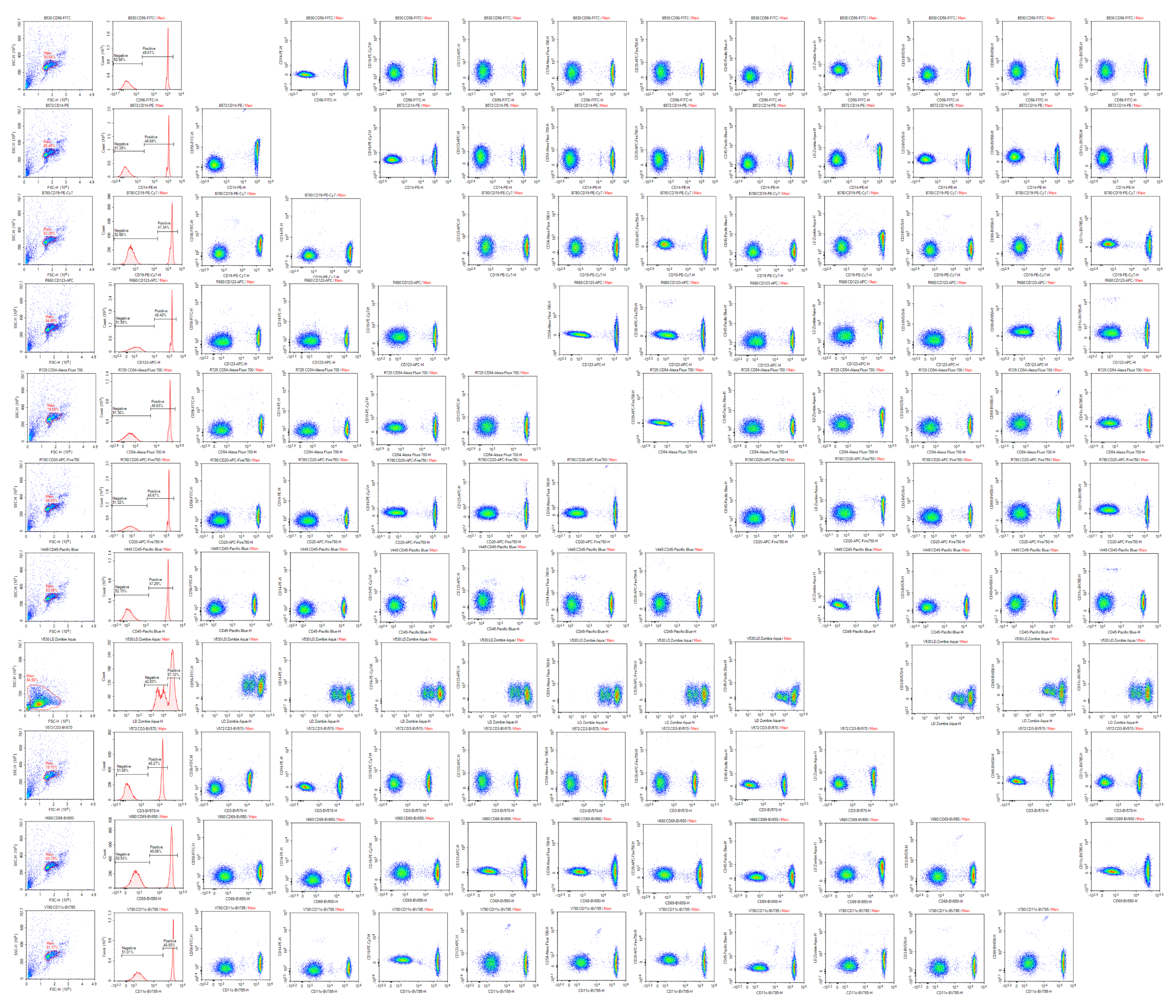


**Figure S3. Representative Immunophenotyping Panel #2 single stain compensation.** A representative replicate of Immunophenotyping Panel #2 single stain controls run with compensation beads and PBMC. Each row of plots is a different single stain control. The first dot plot selects the main population of beads or PBMC. The second plot (histogram) identifies the negative and positive events. The remaining plots in each row show the negative and positive events plotted against each different channel used.


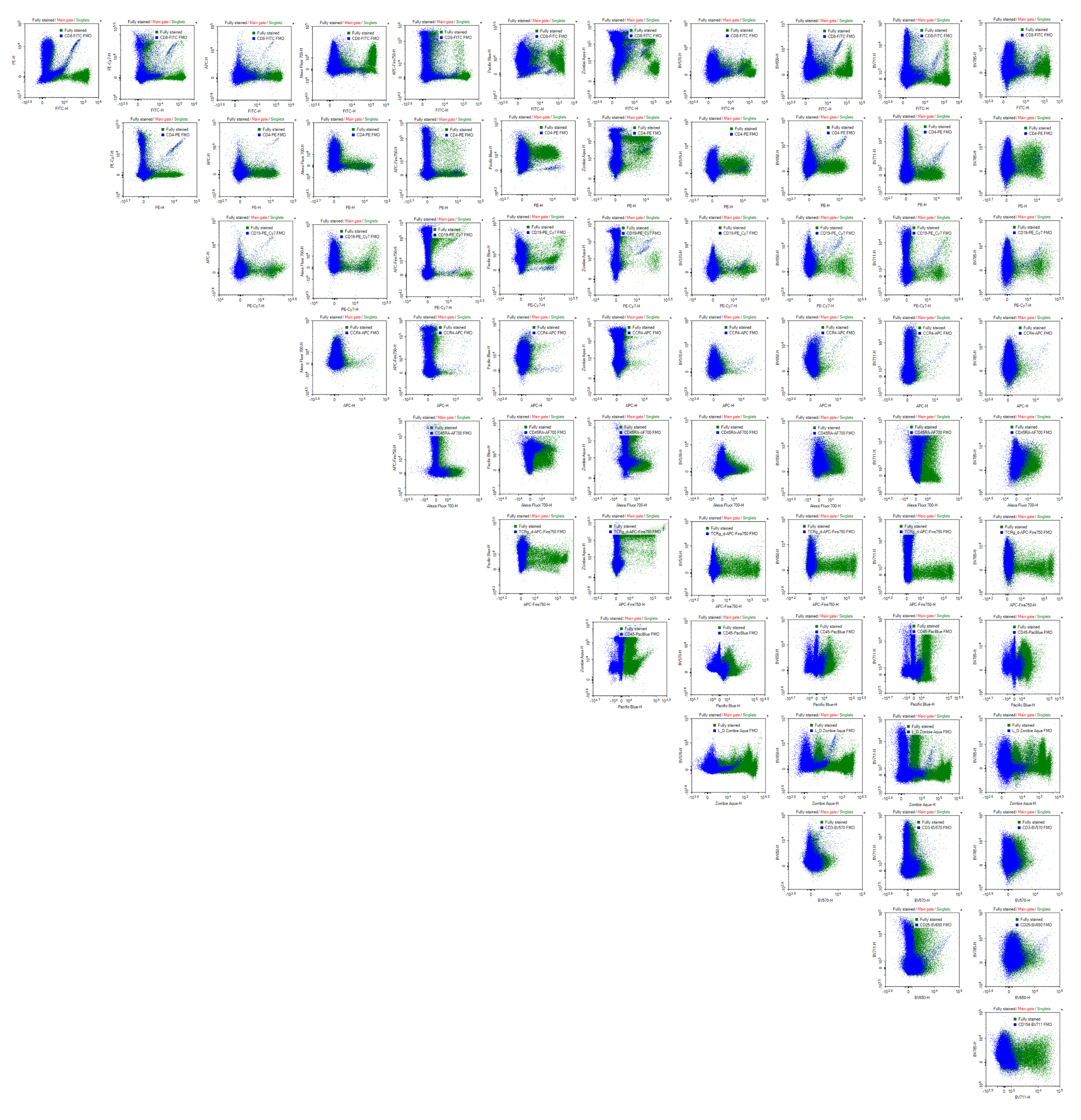


**Figure S4**. **Representative FMO controls for Immunophenotyping Panel #1.** Healthy donor PBMC were activated with PMA/Ionomycin for 24 hours. PBMC samples were stained with all panel antibodies minus one. A PBMC sample was also stained with all labeling antibodies included in the panel. The fully stained PBMC sample (green) was overlayed with each of the FMO controls (blue).


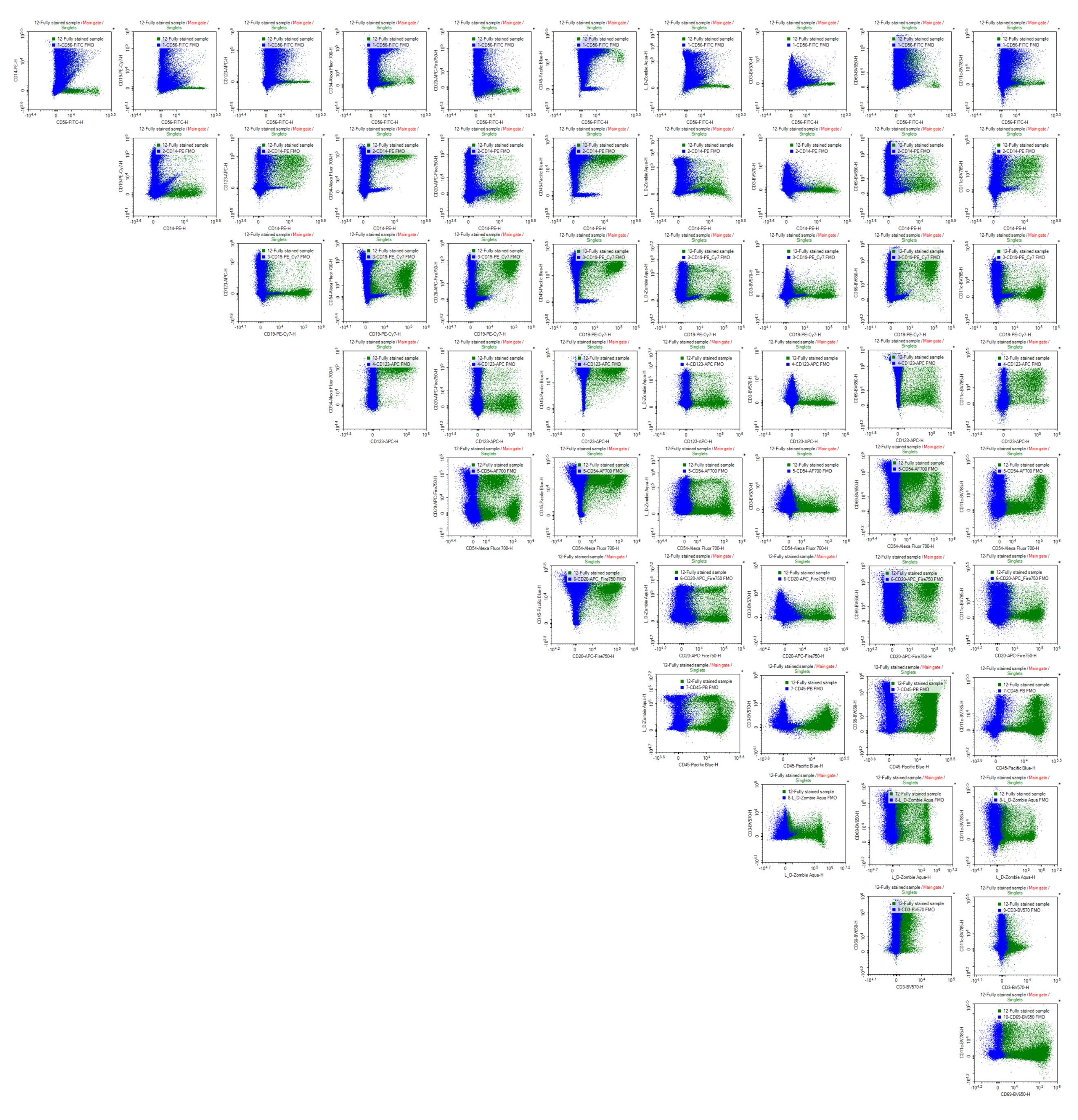


**Figure S5. Representative FMO controls for Immunophenotyping Panel #2.** Healthy donor PBMC were activated with ODN2216/PHA-M for 24 hours. PBMC samples were stained with all panel antibodies minus one. A PBMC sample was also stained with all labeling antibodies included in the panel. The fully stained PBMC sample (green) was overlayed with each of the FMO controls (blue).


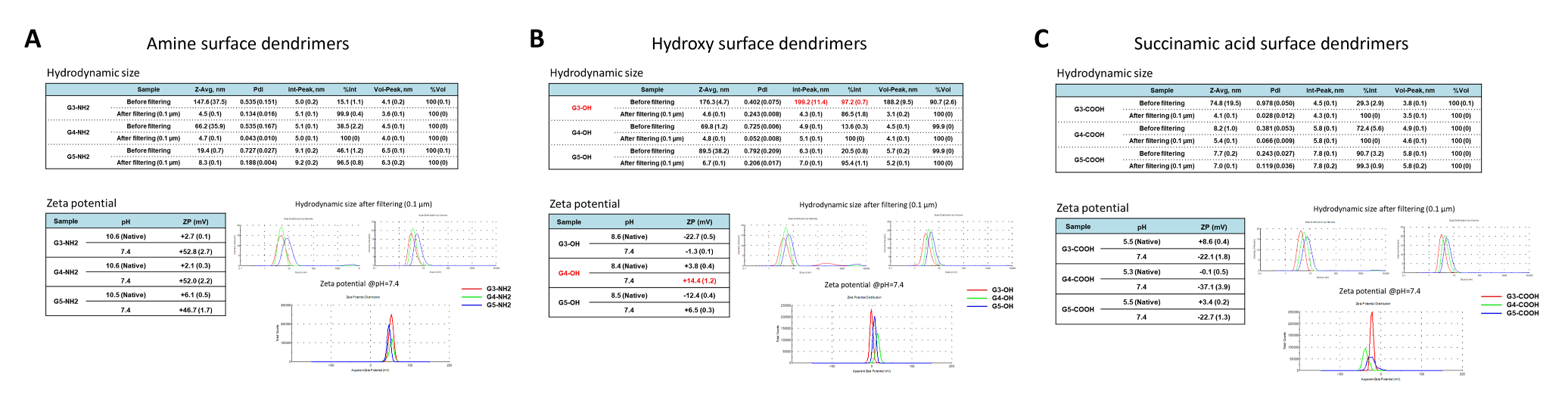


**Figure S6. PCC of Dendritech Dendrimers.** DLS (used to determine hydrodynamic size) and zeta potential were used to characterize **(A)** amine surface **(B)** hydroxy surface **(C)** succinamic acid surface dendrimers.


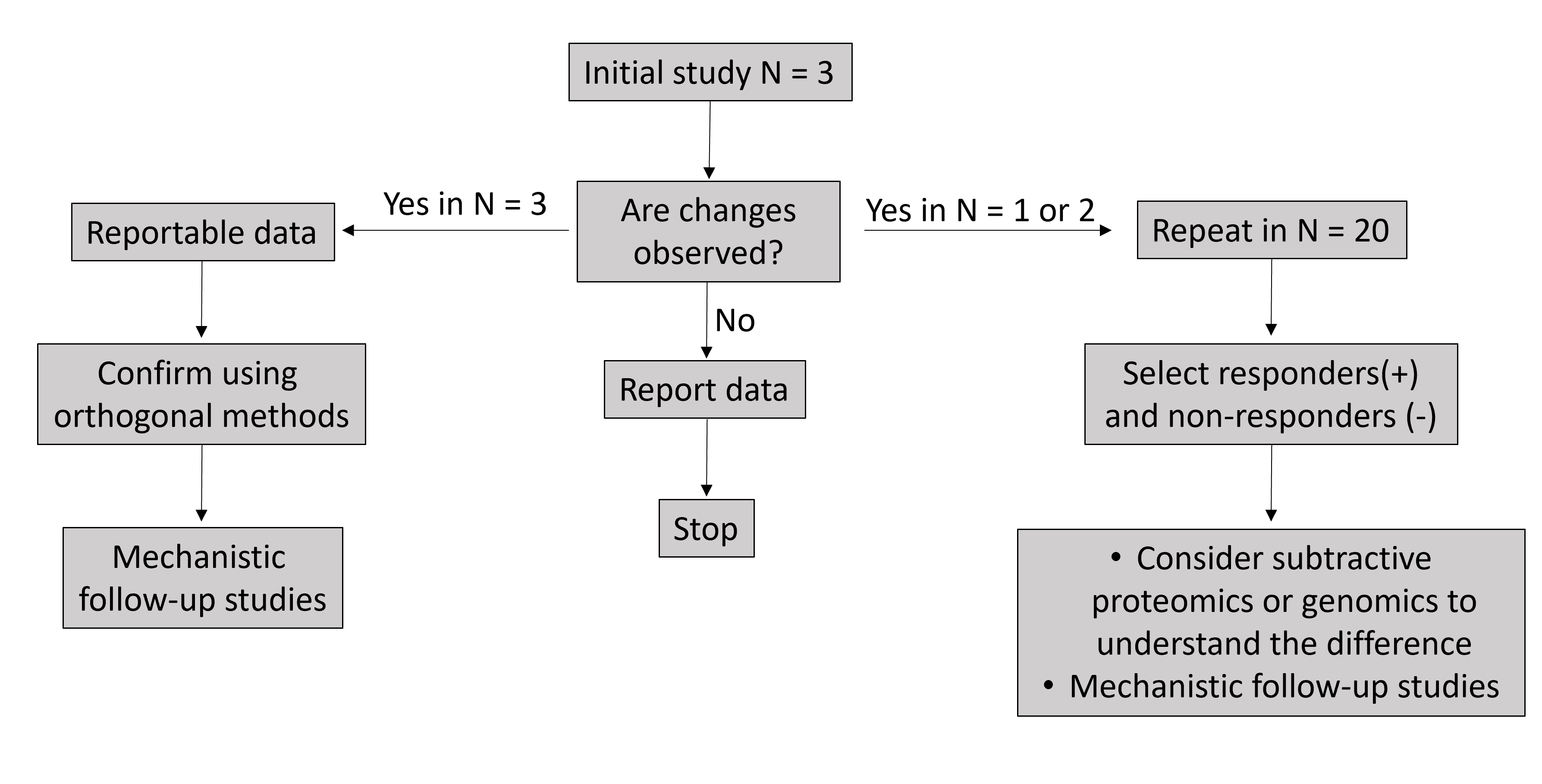


**Figure S7. Decision Tree Example.** This decision tree is designed to help researchers prioritize resources given the various responses seen in different donors where N equals the number of donors used in the study. N=3 is optimal for a pilot study and assay performance qualification; N=10 is recommended for assay validation; N=20 is for follow-up mechanistic studies; a larger number of donors (N=50 or 100) is ideal for statistical analysis.

**Table S1. Endotoxin levels of Dendritech Dendrimers.**

| **Dendrimer** | **Endotoxin EU/mg (spike recovery %)** |
| --- | --- |
| G3-NH2 | <0.05 EU/mg (118) |
| G4-NH2 | 0.0143 EU/mg (71) |
| G5-NH2 | 0.009 EU/mg (121) |
| G5-OH | 0.001 EU/mg (136) |
| G3-COOH | 0.924 EU/mg (62) |
| G4-COOH | 0.979 EU/mg (121) |
| G5-COOH | 0.996 EU/mg (138) |

**Table S2. Mean Difference and P-values for Immunophenotyping Panel #1.** Mean difference of cell population percentages of negative (vehicle) controls and treatments along with the respective p-values. P-values are visually represented in Figure 10A.

| **Mean differences of Cell Population Percentages** | | | | | | | | |
| --- | --- | --- | --- | --- | --- | --- | --- | --- |
| Cell Type | Negative Control G5-COOH | Negative Control G5-OH | Negative Control G5-NH2(G5) | Negative Control G5-NH2(NH2) | Negative Control G4-NH2 | Negative Control G3-NH2 | Dextrose Ctr 1 ug/mL Ambisome | PBS Ctr 1 mg/mL Feraheme |
| Live cells/PBMCs | 2.735 | 1.19166667 | -1.25 | -2.733333333 | -0.14833333 | -0.925 | -1.00333333 | 0.86666667 |
| CD45+ | 0.79333333 | 1.95166667 | 1.58333333 | -0.043333333 | -0.13833333 | 1.20666667 | 1.59333333 | 1.04666667 |
| CD19+ | -0.05333333 | -0.43666667 | -7.68666667 | -4.383333333 | -1.80166667 | -0.05666667 | 0.465 | 0.22833333 |
| CD19+ CD25-/CD154- | 1.98333333 | 1.88166667 | 1.22333333 | 7.653333333 | -2.03 | 6.63 | 1.435 | -0.89666667 |
| CD19+ CD25-/CD154+ | -0.565 | -0.555 | -0.125 | 0.091666667 | -0.04166667 | 0.165 | -1.00333333 | 0.59166667 |
| CD19+ CD25+/CD154+ | -0.37 | -0.365 | 1.83 | -0.4 | 0.69333333 | -0.53166667 | -0.10333333 | 0.05333333 |
| CD19+ CD25+/CD154- | -1.04333333 | -0.96333333 | -2.93 | -7.343333333 | 1.38 | -6.265 | -0.33166667 | 0.25166667 |
| CD3+ | -0.53166667 | 1.01 | -37.9916667 | -27.365 | -20.8516667 | -17.5983333 | 1.34833333 | -0.585 |
| CD8+ | -0.875 | -0.20666667 | -6.95333333 | -4.578333333 | -2.90833333 | -2.79833333 | 0.33 | -0.37166667 |
| CD8+ CD25-/CD154- | 1.68 | 1.685 | 1.57833333 | 1.183333333 | -0.50666667 | 0.21666667 | 0.58166667 | 0.09166667 |
| CD8+ CD25-/CD154+ | -1.49333333 | -1.52666667 | -1.50333333 | -0.988333333 | 0.69333333 | -0.04166667 | -0.55 | -0.01166667 |
| CD8+ CD25+/CD154+ | -0.065 | -0.06333333 | 0.06833333 | 0.001666667 | -0.00833333 | -0.01833333 | -0.025 | -0.075 |
| CD8+ CD25+/CD154- | -0.11833333 | -0.09333333 | -0.14333333 | -0.203333333 | -0.18 | -0.15666667 | -0.00333333 | -0.00333333 |
| CD8+CD45RA+ | -0.44666667 | -0.355 | -3.48 | -0.39 | -0.29333333 | 0.56 | 0.04166667 | -0.06666667 |
| CD4+ | -0.245 | 0.63333333 | -28.17 | -21.28666667 | -16.65 | -13.8316667 | 1.00166667 | -0.51666667 |
| CD4+ CD25-/CD154- | 5.17 | 5.03166667 | 9.23333333 | 6.881666667 | 3.23166667 | 4.34333333 | 2.355 | 0.15333333 |
| CD4+ CD25-/CD154+ | -2.225 | -2.165 | -2.68833333 | -1.538333333 | 1.09 | -0.08833333 | -1.69666667 | 0.3 |
| CD4+ CD25+/CD154+ | -0.335 | -0.32666667 | -0.25 | -0.125 | -0.01833333 | -0.065 | -0.09833333 | -0.11666667 |
| CD4+ CD25+/CD154- | -2.60833333 | -2.54 | -6.29166667 | -5.215 | -4.30333333 | -4.18666667 | -0.565 | -0.33166667 |
| CCR4+ | -1.16 | -1.30166667 | -2.74333333 | -0.348333333 | -1.36166667 | -0.345 | 0.075 | -0.05166667 |
| Tregs | -0.01333333 | -0.01833333 | -0.03333333 | -0.023333333 | -0.01166667 | -0.00833333 | -0.00166667 | 0 |
| CD8-/CD4- | 0.085 | -0.04166667 | -0.81166667 | -0.021666667 | -0.44833333 | -0.38833333 | 0.095 | -0.13166667 |
| TCRg/d | -0.01166667 | 0.005 | -0.00833333 | 0 | 0 | 0 | 0 | 0 |
| **P-values of Mean differences** | | | | | | | | |
| Cell Type | Negative Control G5-COOH | Negative Control G5-OH | Negative Control G5-NH2(G5) | Negative Control G5-NH2(NH2) | Negative Control G4-NH2 | Negative Control G3-NH2 | Dextrose Ctr 1 ug/mL Ambisome | PBS Ctr 1 mg/mL Feraheme |
| Live cells/PBMCs | 0.250200527 | 0.645569936 | 0.420486296 | 0.314529451 | 0.863303699 | 0.410534004 | 0.586944705 | 0.109873476 |
| CD45+ | 0.620314699 | 0.231542425 | 0.354978772 | 0.97732725 | 0.867155442 | 0.041265615 | 0.394465225 | 0.568396195 |
| CD19+ | 0.803677287 | 0.495881579 | 0.000118641 | 0.119794246 | 0.114296453 | 0.946761148 | 0.350834438 | 0.496074857 |
| CD19+ CD25-/CD154- | 0.260019789 | 0.298963682 | 0.384685758 | 0.250310164 | 0.635734309 | 0.274661203 | 0.148235443 | 0.482427137 |
| CD19+ CD25-/CD154+ | 0.124661996 | 0.141920707 | 0.601666329 | 0.768525161 | 0.919298975 | 0.26823092 | 0.22750011 | 0.621092858 |
| CD19+ CD25+/CD154+ | 0.162580099 | 0.225926079 | 0.352536488 | 0.387483989 | 0.425576529 | 0.398523117 | 0.157222766 | 0.51975982 |
| CD19+ CD25+/CD154- | 0.386419572 | 0.436447987 | 0.044750854 | 0.257592004 | 0.719073844 | 0.262452932 | 0.046558204 | 0.35111806 |
| CD3+ | 0.789010227 | 0.514969303 | 0.000301891 | 0.306014343 | 0.127970729 | 0.367020764 | 0.288859563 | 0.727881221 |
| CD8+ | 0.137979842 | 0.292594109 | 0.034606352 | 0.526954034 | 0.311887921 | 0.582761196 | 0.469361473 | 0.472928109 |
| CD8+ CD25-/CD154- | 0.15234178 | 0.144832546 | 0.078610225 | 0.027782131 | 0.459543385 | 0.469117934 | 0.15192029 | 0.883220859 |
| CD8+ CD25-/CD154+ | 0.205306426 | 0.187914541 | 0.125103545 | 0.039006434 | 0.319847275 | 0.871500532 | 0.157839852 | 0.983699184 |
| CD8+ CD25+/CD154+ | 0.275163052 | 0.252744128 | 0.281365008 | 0.9641252 | 0.422649731 | 0.235447148 | 0.285714286 | 0.357863219 |
| CD8+ CD25+/CD154- | 0.242060072 | 0.308167304 | 0.310785914 | 0.124857588 | 0.083824123 | 0.064016115 | 0.825922344 | 0.666666667 |
| CD8+CD45RA+ | 0.201850543 | 0.177029365 | 0.115071341 | 0.390116055 | 0.290610873 | 0.366702473 | 0.603474207 | 0.331099938 |
| CD4+ | 0.874981964 | 0.542726568 | 0.007063034 | 0.261739997 | 0.107421364 | 0.31189531 | 0.218752166 | 0.648314439 |
| CD4+ CD25-/CD154- | 0.079863095 | 0.102599975 | 0.004644069 | 0.002484923 | 0.079565851 | 0.014593867 | 0.149862428 | 0.902501117 |
| CD4+ CD25-/CD154+ | 0.176267194 | 0.202433087 | 0.091333649 | 0.015890241 | 0.114128496 | 0.877465409 | 0.119614959 | 0.807957141 |
| CD4+ CD25+/CD154+ | 0.029257329 | 0.032001385 | 0.01904196 | 0.000532907 | 0.440838575 | 0.030138774 | 0.112455758 | 0.469148286 |
| CD4+ CD25+/CD154- | 0.193598989 | 0.236397234 | 0.054426862 | 0.00958272 | 0.018309848 | 0.008360962 | 0.260183221 | 0.180736908 |
| CCR4+ | 0.420999121 | 0.332846248 | 0.208781304 | 0.307692683 | 0.158032129 | 0.449068506 | 0.422649731 | 0.422649731 |
| Tregs | 0.48993863 | 0.368066511 | 0.241901956 | 0.033908217 | 0.422649731 | 0.559774547 | 0.422649731 | #NUM! |
| CD8-/CD4- | 0.63447199 | 0.662869419 | 0.098894126 | 0.949120445 | 0.160427904 | 0.421978573 | 0.746003529 | 0.695610774 |
| TCRg/d | 0.249521226 | 0.422649731 | 0.299859958 | #NUM! | #NUM! | #NUM! | #NUM! | #NUM! |

**Table S3**. **Mean Difference and P-values for Immunophenotyping Panel #2.** Mean difference of cell population percentages of negative (vehicle) controls and treatments along with the respective p-values. P-values are visually represented in Figure 10B.

| **Mean differences of Cell Population Percentages** | | | | | | | | |
| --- | --- | --- | --- | --- | --- | --- | --- | --- |
| Cell Type | Negative Ctr G5-COOH | Negative Ctr G5-OH | Negative Ctr G5-NH2(G5) | Negative Ctr G5-NH2(NH2) | Negative Ctr G4-NH2 | Negative Ctr G3-NH2 | Dextrose Ctr 1 ug/mL Ambisome | PBS Ctr 1 mg/mL Feraheme |
| Live_M | 1.52 | -0.43166667 | -23.2366667 | -21.745 | -6.83666667 | -12.185 | -0.32 | -1.59166667 |
| CD14+ | 7.126666667 | -3.735 | -2.56166667 | -1.20166667 | 0.47666667 | 3.78166667 | 3.53666667 | -6.04666667 |
| CD14+ CD69-/CD54- | 0.668333333 | 1.93 | 5.01333333 | 16.65 | 0.72666667 | 14.6233333 | -0.045 | 11.3716667 |
| CD14+ CD69-/CD54+ | 2.27 | 2.04 | 23.6266667 | 1.2225 | 3.40833333 | 7.64333333 | -2.88 | -13.4616667 |
| CD14+ CD69+/CD54+ | -2.806666667 | -3.80166667 | -28.3433333 | -18.2275 | -9.37666667 | -8.93666667 | 3.53333333 | -10.085 |
| CD14+ CD69+/CD54- | -0.135 | -0.17 | -0.295 | 0.36 | 5.24666667 | -13.3233333 | -0.60833333 | 12.175 |
| Live_L | 0.7 | 1.60333333 | -0.755 | -1.15 | 0.16833333 | -0.29 | 0.18333333 | 3.08833333 |
| CD123+_L (pDCs) | 0.231666667 | 0.01166667 | -0.27333333 | -0.26166667 | -0.295 | -0.17666667 | -0.055 | 0.00833333 |
| CD123+_L (pDCs) CD69-/CD54- | -1.408333333 | 2.96333333 | 10.7716667 | 7.5675 | 3.8675 | 14.005 | 2.37166667 | 9.275 |
| CD123+_L (pDCs) CD69-/CD54+ | 1.623333333 | -3.20833333 | -10.965 | -0.97 | -2.72 | -6.2625 | -0.19666667 | -6.25833333 |
| CD123+_L (pDCs) CD69+/CD54+ | -0.195 | 0.20833333 | 0.05666667 | -1.06 | 1.07 | -4.6625 | -1.01833333 | -1.65 |
| CD123+_L (pDCs) CD69+/CD54- | -0.021666667 | 0.03333333 | 0.135 | -5.545 | -2.2225 | -3.0825 | -1.15166667 | -1.36833333 |
| CD11c+_L (mDCs) | 0.261666667 | 0.495 | 0.25666667 | -0.43166667 | -0.065 | -0.06666667 | -0.435 | -0.155 |
| CD11c+_L (mDCs) CD69-/CD54- | -0.8 | 2.31833333 | -4.13833333 | 4.43666667 | -4.21333333 | 0.31833333 | -2.77666667 | 5.85666667 |
| CD11c+_L (mDCs) CD69-/CD54+ | 0.033333333 | -2.79333333 | -2.87 | -9.915 | -12.4716667 | -10.83 | 2.14833333 | -2.245 |
| CD11c+_L (mDCs) CD69+/CD54+ | -0.023333333 | -0.17333333 | 2.21166667 | 2.93166667 | 4.80166667 | 1.98666667 | 0.21833333 | -1.60333333 |
| CD11c+_L (mDCs) CD69+/CD54- | 0.79 | 0.65333333 | 4.80333333 | 2.54333333 | 11.885 | 8.52833333 | 0.41166667 | -2.01166667 |
| CD3-CD56+ (NK cells) | -0.171666667 | 0.545 | -1.64166667 | -1.78833333 | -2.28666667 | -2.275 | 0.44666667 | -0.15333333 |
| CD3-CD56+ (NK cells) CD69-/CD54- | 4.521666667 | 5.43166667 | -3.515 | -6.46666667 | -10.1366667 | -2.33666667 | -0.125 | -0.28833333 |
| CD3-CD56+ (NK cells) CD69-/CD54+ | -3.998333333 | -4.93833333 | 1.95333333 | 6.56833333 | 8.905 | 1.78333333 | 0.16166667 | 0.495 |
| CD3-CD56+ (NK cells) CD69+/CD54+ | -0.273333333 | -0.3 | 0.59333333 | 0.21166667 | 0.60666667 | 0.14 | 0.07166667 | -0.03 |
| CD3-CD56+ (NK cells) CD69+/CD54- | -0.253333333 | -0.19333333 | 0.96666667 | -0.31666667 | 0.62666667 | 0.415 | -0.10833333 | -0.17333333 |
| CD3+CD56+ (NK T cells) | 0.098333333 | 0.08166667 | -0.12 | -0.15 | -0.21 | -0.205 | 0.01 | 0.00166667 |
| **P-values of Mean differences** | | | | | | | | |
| Cell Type | Negative Ctr G5-COOH | Negative Ctr G5-OH | Negative Ctr G5-NH2(G5) | Negative Ctr G5-NH2(NH2) | Negative Ctr G4-NH2 | Negative Ctr G3-NH2 | Dextrose Ctr 1 ug/mL Ambisome | PBS Ctr 1 mg/mL Feraheme |
| Live_M | 0.13789301 | 0.56719754 | 0.035145234 | 0.093714021 | 0.246603131 | 0.058096351 | 0.557288621 | 0.757077444 |
| CD14+ | 0.218090719 | 0.645126824 | 0.914322234 | 0.826024085 | 0.944301014 | 0.504853881 | 0.434221558 | 0.173323963 |
| CD14+ CD69-/CD54- | 0.431315504 | 0.393905817 | 0.254548129 | 0.358460004 | 0.942226037 | 0.376768386 | 0.914918736 | 0.480605052 |
| CD14+ CD69-/CD54+ | 0.762835979 | 0.754871101 | 0.060068414 | 0.917732586 | 0.63899316 | 0.188724571 | 0.531813863 | 0.221230639 |
| CD14+ CD69+/CD54+ | 0.71791053 | 0.56849073 | 0.06770172 | 0.04072087 | 0.1814379 | 0.177658217 | 0.381327003 | 0.376328682 |
| CD14+ CD69+/CD54- | 0.40708247 | 0.584127028 | 0.766879743 | 0.105136913 | 0.397041669 | 0.422289861 | 0.794306321 | 0.072659402 |
| Live_L | 0.70286521 | 0.165234314 | 0.227069365 | 0.130172092 | 0.009946653 | 0.223183321 | 0.11017913 | 0.389434137 |
| CD123+_L (pDCs) | 0.489381077 | 0.931598994 | 0.306366316 | 0.169591087 | 0.082112674 | 0.368712024 | 0.225403331 | 0.852107248 |
| CD123+_L (pDCs) CD69-/CD54- | 0.719559589 | 0.488271688 | 0.280356777 | 0.532690023 | 0.551332483 | 0.33517837 | 0.385931032 | 0.296125367 |
| CD123+_L (pDCs) CD69-/CD54+ | 0.729190881 | 0.497318899 | 0.232602762 | 0.760449205 | 0.50233193 | 0.627650604 | 0.961861218 | 0.360039409 |
| CD123+_L (pDCs) CD69+/CD54+ | 0.784572029 | 0.522605099 | 0.944175061 | 0.836260395 | 0.574477583 | 0.219261968 | 0.299571183 | 0.218268427 |
| CD123+_L (pDCs) CD69+/CD54- | 0.94382077 | 0.961069301 | 0.856612012 | 0.210669999 | 0.127867792 | 0.490569286 | 0.43727272 | 0.296940345 |
| CD11c+_L (mDCs) | 0.028473531 | 0.242610587 | 0.845277148 | 0.055643473 | 0.70767443 | 0.529379195 | 0.088075698 | 0.204605091 |
| CD11c+_L (mDCs) CD69-/CD54- | 0.903104031 | 0.640851701 | 0.606569467 | 0.620049866 | 0.81565112 | 0.984518948 | 0.029891341 | 0.53595593 |
| CD11c+_L (mDCs) CD69-/CD54+ | 0.996225881 | 0.618088992 | 0.585970717 | 0.232099527 | 0.203706301 | 0.33905267 | 0.129254187 | 0.694471115 |
| CD11c+_L (mDCs) CD69+/CD54+ | 0.866284074 | 0.104313192 | 0.298032441 | 0.300903172 | 0.043027554 | 0.203107426 | 0.210140798 | 0.392612129 |
| CD11c+_L (mDCs) CD69+/CD54- | 0.629481423 | 0.522177854 | 0.199223273 | 0.413737484 | 0.307678103 | 0.39347444 | 0.289604882 | 0.31359989 |
| CD3-CD56+ (NK cells) | 0.632646218 | 0.335904727 | 0.249151214 | 0.094671421 | 0.11543313 | 0.119521756 | 0.256261084 | 0.022382373 |
| CD3-CD56+ (NK cells) CD69-/CD54- | 0.339235144 | 0.346887873 | 0.439252652 | 0.187257751 | 0.159690797 | 0.60931233 | 0.737722154 | 0.631272525 |
| CD3-CD56+ (NK cells) CD69-/CD54+ | 0.36770082 | 0.367938649 | 0.292306917 | 0.166901536 | 0.20306578 | 0.730438274 | 0.426537247 | 0.345546621 |
| CD3-CD56+ (NK cells) CD69+/CD54+ | 0.311374035 | 0.338699929 | 0.38884638 | 0.238893016 | 0.28086269 | 0.597888154 | 0.490794322 | 0.427922446 |
| CD3-CD56+ (NK cells) CD69+/CD54- | 0.20293118 | 0.147933659 | 0.63615713 | 0.114255209 | 0.503000167 | 0.431848803 | 0.554407626 | 0.474948328 |
| CD3+CD56+ (NK T cells) | 0.597969125 | 0.476556665 | 0.244819869 | 0.063414188 | 0.03712938 | 0.019258495 | 0.183503419 | 0.74180111 |
